# Dynamic changes in gene expression and signalling during trophoblast development in the horse

**DOI:** 10.1530/REP-18-0270

**Published:** 2018-07-10

**Authors:** Jordan E Read, Victoria Cabrera-Sharp, Victoria Offord, Samantha M Mirczuk, Steve P Allen, Robert C Fowkes, Amanda M de Mestre

**Affiliations:** 1 Department of Comparative Biomedical Sciences The Royal Veterinary College, Hertfordshire, UK; 2 Research Support Office The Royal Veterinary College, London, UK

## Abstract

Equine chorionic girdle trophoblast cells play important endocrine and immune functions critical in supporting pregnancy. Very little is known about the genes and pathways that regulate chorionic girdle trophoblast development. Our aim was to identify genes and signalling pathways active *in vivo* in equine chorionic girdle trophoblast within a critical 7-days window. We exploited the late implantation of the equine conceptus to obtain trophoblast tissue. An Agilent equine 44K microarray was performed using RNA extracted from chorionic girdle and chorion (control) from equine pregnancy days 27, 30, 31 and 34 (*n* = 5), corresponding to the initiation of chorionic girdle trophoblast proliferation, differentiation and migration. Data were analysed using R packages limma and maSigPro, Ingenuity Pathway Analysis and DAVID and verified using qRT-PCR, promoter analysis, western blotting and migration assays. Microarray analysis showed gene expression (absolute log FC >2, FDR-adjusted *P* < 0.05) was rapidly and specifically induced in the chorionic girdle between days 27 and 34 (compared to day 27, day 30 = 116, day 31 = 317, day 34 = 781 genes). Pathway analysis identified 35 pathways modulated during chorionic girdle development (e.g. FGF, integrin, Rho GTPases, MAPK) including pathways that have limited description in mammalian trophoblast (e.g. IL-9, CD40 and CD28 signalling). Rho A and ERK/MAPK activity was confirmed as was a role for transcription factor ELF5 in regulation of the CGB promoter. The purity and accessibility of chorionic girdle trophoblast proved to be a powerful resource to identify candidate genes and pathways involved in early equine placental development.

## Introduction

Mammalian placental development requires a highly regulated series of cellular processes that transform a single layer of trophectoderm into a complex membrane that interacts intimately with maternal tissues. Although the anatomical structure of mature mammalian placentae is highly variable between species, the processes that lead to its formation are remarkably similar (Noronha & Antczak 2010, Soncin *et al.* 2015). Trophoblast cells that give rise to the placenta must undergo a period of rapid proliferation, followed by cell movement, differentiation, migration and in some species also invasion. Once trophoblast penetrate the endometrial epithelium, their immunomodulatory behaviour becomes critical to the prevention of immune-mediated pregnancy loss (Noronha & Antczak 2010, de Mestre *et al.* 2011, Moffett *et al.* 2017). These cellular processes are difficult to study in human pregnancy due to the limited availability of tissues at key consecutive developmental stages. Consequently, much work has focused on rodent models of placentation (Underhill & Robins 2016), *in vitro* models of human pregnancy (Sibley 2017), together with comparative models of placentation (Imakawa *et al.* 2017) that are both informative for understanding basic mammalian processes as well as being relevant to the species itself. Collectively these studies have revealed a number of genes and signalling pathways involved in placental development (Soncin *et al.* 2015, Gupta *et al.* 2016, Haeger *et al.* 2016) but exactly how important each of these pathways are for the multiple/overlapping processes that are occurring during development and whether these pathways are all active *in vivo* is less clear.

The early equine conceptus is unique in that it remains in a spherical shape surrounded by a glycoprotein capsule for the first 21 days of gestation. It is only after this capsule is lost that placental development begins in earnest (Allen & Wilsher 2009). Further, the late implantation in the horse (around day 40) means it is remarkably accessible and remains essentially separate to maternal tissues throughout early placental development. This provides distinct advantages over other species as it allows for isolation of pure populations of trophoblast cells with minimal manipulation (Noronha & Antczak 2010). By day 30 of gestation, the horse placenta is comprised of two main membranes. First, the allantochorion that undergoes rapid growth to form the diffuse epitheliochorial placenta of the horse primarily involved in nutritional exchange and later endocrine support of the pregnancy (Allen & Wilsher 2009). Second, the unique chorionic girdle which gives rise to the endometrial cups that secrete equine chorionic gonadotrophin (eCG) (Antczak *et al.* 2013) and modulate immunity to foetally derived tissues (de Mestre *et al.* 2010, 2011). Around day 27 of gestation, the trophoblast cells of the chorionic girdle begin to rapidly proliferate leading to multiple layers of trophoblast cells. Around day 30 of gestation chorionic girdle trophoblast cells receive a signal to terminally differentiate from uninucleate cells to mature eCG-secreting binucleate cells (Cabrera-Sharp *et al.* 2014) – a process which is close to complete by days 34–36 of gestation. As the trophoblast cells differentiate they acquire a highly invasive phenotype and penetrate the endometrial luminal epithelium and migrate down the glands. Motility then ceases, cells enlarge and form what can be seen as mature endometrial cup trophoblast. The molecular mechanisms that regulate the development of the chorionic girdle, including the co-ordinated processes of proliferation, differentiation, cell movement and cell migration are poorly understood.

We recently identified bone morphogenetic protein (BMP) 4 signalling as a key regulator of trophoblast differentiation in the horse (Cabrera-Sharp *et al.* 2014). Whilst BMP4 was able to drive terminal differentiation and eCG secretion by the cells, not all cells differentiated in response to the ligand. This suggests other signals are also required to drive chorionic development. Expression of additional growth factor receptors during chorionic girdle development has also been shown (Stewart *et al.* 1994, 1995, Allen *et al.* 2007, 2017) although the functional importance and the activity of the associated molecular pathways has not been investigated. Previous studies have used equine-specific microarrays to study gene expression and/or imprinting in the placenta between days 8 and 14 and at day 34 (Klein & Troedsson 2011, Brosnahan *et al.* 2012, Wang *et al.* 2013, Iqbal *et al.* 2014). These studies have found vital insights into the role of interleukin 22 (IL-22) in trophoblast invasive capacity (Brosnahan *et al.* 2012) and to factors likely to be important in the process of maternal recognition of pregnancy (Klein & Troedsson 2011). An understanding of the gene changes in the chorionic girdle during the time of trophoblast proliferation, differentiation, invasion and induction of eCG expression and secretion would offer key advances in the knowledge of these complex processes.

In this present study, we exploited the late implantation of the equine conceptus to obtain trophoblast tissue between days 27 and 34 of pregnancy from matched mare and stallion pairs. We used whole transcriptome profiling in order to measure and compare gene expression in chorionic girdle trophoblast and adjacent regressing chorion at pregnancy day 27 (initiation of proliferation and prior to differentiation), day 30 (initiation of differentiation), day 31 (consolidation of differentiation and movement of cells) and day 34 (when the majority of the trophoblast cells have terminally differentiated into binucleate eCG-secreting trophoblast and have started to obtain invasive qualities and immunomodulatory capacities). Differentially expressed genes were then identified to determine functions and signalling pathways whose activity was modulated over this critical period of trophoblast development. A selection of genes and pathways were subsequently validated.

## Materials and methods

### Animals and tissue collection

The study was approved by the Ethics Committee of the Royal Veterinary College and Home Office (PPL70/6944), and all animal procedures were performed in accordance with the Animals (Scientific Procedures) Act 1986 guidelines set by the Home Office, United Kingdom. Five mares (*Equus caballus*) aged between 3 and 7 years were of Dartmoor or Welsh breed and maintained in a paddock on grass and supplemented with hay over the winter. The reproductive cycle was manipulated using prostaglandin F2, and pregnancies were established as previously described (de Mestre *et al*. 2011) using semen from two stallions, standard artificial insemination and with ovulation induced using either 1500 IU hCG (Chorulon, MSD Animal Health, Milton Keynes, UK) intravenously or 2.1 mg Ovuplant (Dechra Veterinary Products, Shrewsbury, UK) subcutaneously. Ovulation was confirmed (day 0) and then pregnancies were monitored biweekly and on the day of isolation using transrectal ultrasonographic evaluation of the reproductive tract. Only those conceptuses confirmed to have a normal growth rate and normal anatomical development were included in the study. The same mare and stallion pairs (*n* = 5) (Supplementary Table 1 shows exceptions to this, see section on supplementary data given at the end of this article) were used to generate multiple conceptuses that were recovered at day 27, 30, 31 and 34 of pregnancy via non-surgical uterine lavage as previously described (de Mestre *et al.* 2008). Additional mare and stallion combinations (*n* = 3) generated conceptus tissue that was subsequently used for qRT-PCR and western blotting. Conceptuses were flushed into sterile phosphate buffered solution supplemented with 2× penicillin–streptomycin (5000 U/mL, Invitrogen Gibco) and placed immediately onto ice for transport to the laboratory. Conceptuses were dissected in PBS-2xPenStrep into components, chorionic girdle, chorion, allantochorion, yolk sac and foetus using a dissecting microscope (Zeiss). Dissected tissue was immediately snap-frozen in liquid nitrogen and stored at −80°C until RNA isolation or western blotting was performed.

### Isolation and qualitative and quantitative analysis of RNA

Total RNA was isolated from snap-frozen equine conceptus tissue, following homogenisation by QIAshredder (Qiagen), using a Qiagen RNeasy kit (Qiagen) as directed by the manufacturer. RNA was additionally treated with DNase on a column (Qiagen) as described by the manufacturer. RNA concentration and purity was determined by spectrophotometry, using the Nanodrop ND1000 spectrophotometer. The mean 260/280 ratio was 2.10 (range 2.02–2.16). RNA quality was additionally assessed using Agilent 2100 Bioanalyser. The mean and median RNA integrity number was 9.1 (range 8–9.9) (Supplementary Fig. 1). Samples were stored at −80°C.

### Microarray analysis

A 44K probe, single colour, equine Agilent Microarray (cyanine 3-CTP; Agilent Technologies UK Ltd) was carried out by Dr. Lucille Rainbow, at the Centre for Genomic Research, The University of Liverpool. The above described DNase-treated RNA samples were submitted for analysis. Using RNA spike-in kits (Agilent Technologies UK Ltd) and via the manufacturer’s instructions, 50 ng of RNA sample was spiked with quality controls and run. cRNA was synthesised and amplified using the low input quick amp labelling kit via the manufacturer’s instructions (Agilent Technologies UK Ltd.). A specific T7 RNA polymerase blend was used to generate fluorescent cRNA via incorporation of Cyanine 3-CTP. cRNA was purified with the RNeasy mini spin column kit (Qiagen), following the manufacturer’s protocol and concentration measured on the NanoDrop ND-1000 Spectrophotometer (Thermo Fisher Scientific). cRNA was hybridised to the microarray chip using the gene expression hybridisation kit (Agilent Technologies UK Ltd.), using the manufacturer’s instructions and then hybridised for 17 h at 65°C and 10 g using the Tecan HS Pro hybridisation station (Tecan group Ltd, Reading, UK). Microarray chips were washed with wash buffer and scanned by the Agilent DNA microarray scanner G2505C (Agilent Technologies UK Ltd). Samples were subjected to Agilent quality control measures, which all entities passed. Data have been submitted to NCBI Gene Expression Omnibus (GSE113072).

Following Principal Component Analysis (PCA) (Supplementary Fig. 2), a subset of two arrays (out of the total of 40) were removed. These two samples were subsequently shown by qRT-PCR (data not shown) to express significantly aberrant levels of well-known markers of chorionic girdle cells such as chorionic gonadotrophin A (*CGA*), chorionic gonadotrophin B (*CGB*) and glial cells missing 1 (*GCM1*). Pre-processing steps of background correction and between-array normalisation were performed prior to analysis using the software packages R (http://www.r-project.org) and Bioconductor (http://www.bioconductor.org). Differential expression analysis was performed using the R package limma (Ritchie *et al.* 2015). Pre-processing steps of background correction and between-array normalisation were implemented prior to analysis with differentially expressed genes identified using a linear model fit (lmFit) and an empirical Bayes method (eBayes). Genes with an absolute log fold change >2 and false discovery rate (FDR)-adjusted *P* < 0.05 were considered significant. Cluster gene analysis was performed using maSigPro (Conesa & Nueda 2017) to group genes based on their expression profile. The cut-off criteria used in maSigPro were fold change >2, *R* ^2^ > 0.7 and *P* value of >0.01. DAVID Bioinformatics Resources 6.7 (Huang *et al.* 2009) functional annotation tool was used to evaluate signalling pathways within gene clusters. Gene lists were input to DAVID, selected to compare against ‘All species’ and gene names defined as ‘Official_gene_symbol’. Output signalling pathways in KEGG and PANTHER databases were considered, with parameters set to include pathways containing >5 genes with *P* < 0.01 (Fishers Exact test).

The normalised intensity data for chorionic girdle tissue only was also imported into Ingenuity Pathway Analysis (IPA) V119043121 software (Qiagen) for analysis of activation of canonical pathways and regulator effect networks. For analysis in IPA, one-way ANOVA (*P* < 0.05) applied across the timecourse was used to identify gene changes and pathway activation within the chorionic girdle. Output data were represented for canonical pathway activation and as summary lists of genes involved in effector networks of interest.

### Multiplex qRT-PCR

Eight genes were selected from the array to fit the following criteria: upregulated or downregulated with a fold change 2–5 or >20 in chorionic girdle between day 27 and day 34. As a control, Nuclear RNA Export Factor 1 (*NXF1*), a gene that remained constant in expression in the chorionic girdle between days 27 and 34, was also assessed. Primers were designed using GeXP eXpress Profile software in accordance to manufacturer instructions (Beckman Coulter, High Wycombe, UK), using mRNA sequences obtains from Ensembl genome browser (http://www.ensembl.org) (Supplementary Table 2). Target-specific reverse transcription and PCR amplification was performed as previously described (Hu *et al*. 2012, Xie *et al*. 2012, Staines *et al*. 2016) and in accordance to manufacturer’s instructions (Beckman Coulter). In brief, a reverse transcription master mix was prepared as detailed in the GeXP starter kit (Beckman Coulter) and performed using a G-storm thermal cycler, using the programme protocol 48°C (1 min), 42°C (60 min), and 95°C (5 min). From this an aliquot was of each reverse transcription reaction was added to GenomeLab kit PCR master mix (Beckman Coulter) and Thermo-Start Taq DNA polymerase (Thermo Fisher Scientific). PCR reaction was again performed using G-storm thermal cycler with a 95°C activation step 10 min, followed by 35 cycles of 94°C (30 s), 55°C (30 s) and 70°C (60 s). Products were separated and quantified using CEQ 8000 Genetic Analysis System and GenomeLab Fragment Analysis software (Beckman Coulter). Genes of interest were normalised against the geo-mean of the two housekeeping genes, glyceraldehyde 3-phosphate dehydrogenase (*GAPDH*) and succinate dehydrogenase complex, subunit A (*SDHA*), as previously described (Vandesompele *et al.* 2002).

### Western blotting

Tissues were ground and lysed on ice in 200 µL lysis buffer containing: 150 mM sodium chloride (Sigma Aldrich, Haverhill, UK), 1.0% (v/v) Nonidet P-40 (Sigma Aldrich), 0.5% (w/v) sodium deoxycholate (Sigma Aldrich), 0.1% (w/v) Sodium dodecyl sulphate (Sigma Aldrich), 50 mM Tris, pH 8.0 (Sigma Aldrich), 1 mM phenylmethylsulfonyl fluoride (Sigma Aldrich, added at time of use). Protein concentration was determined using Bradford assay (Bio-Rad Laboratories) and read using a Tecan infinite pro 200 plate reader, with Magellan 7 software (Tecan group Ltd.). Samples were stored at −20°C until use.

To assess total and phospho p44/42 extracellular signal regulated kinase (ERK1) protein expression, 50 µg of protein were loaded per well and separated by SDS-PAGE on a 10% (wt/vol) polyacrylamide gel before being transferred to a polyvinylidene difluoride membrane (GE Healthcare Life Sciences) using a Mini-PROTEAN Tetra cell wet transfer unit (Bio-Rad Laboratories). The membranes were blocked in Tris-buffered saline-Tween 20 containing 5% (wt/vol) nonfat milk (Marvel, Premier Foods Group, London, UK) for a total of 3 h. Membranes were incubated overnight at 4°C in a 1:1000 dilution of rabbit anti-human p44/42 total ERK or Rabbit anti-human p44/42 phospho-ERK (Cell Signalling Technology, Leiden, The Netherlands) polyclonal antibodies each in Tris-buffered saline-Tween 20 containing 5% (wt/vol) nonfat milk. Membranes were incubated with a 1:10,000 dilution of goat anti-rabbit IgG secondary antibody conjugated to horseradish peroxidase (Sigma) in Tris-buffered saline-Tween 20 containing 5% (wt/vol) nonfat milk. Proteins were visualised by incubating with ECL plus detection reagents (PerkinElmer) and exposed onto Amersham hyperfilm ECL (GE Healthcare Lifesciences). As a loading control, membranes were stripped and reprobed for β-actin using a monoclonal mouse β-actin antibody (Sigma) at a dilution of 1:10,000.

### Cloning of ELF5 and transactivation experiments

Full length equine ELF5 was cloned into the pCMV-Myc expression vector for co-transfection experiments. ELF5 was amplified by RT-PCR from equine day 34 chorionic girdle cDNA using primers ELF5F CCATGGTACCATGATGTTGGACTCAGTGAC and ACGGTCTAGATCATAGCTTGTCCTCCTGCC. The pCMV-Myc vector and ELF5 PCR product were subjected to digest with XbaI and KpnI restriction and gel purified before ligation into pCMV-myc. Successful cloning was assessed by analytical digest and Sanger sequencing. Truncated promoter constructs of specific lengths of the *CG/LHB* promoter were constructed via PCR from equine genomic DNA as previously described (Read *et al.* 2018). The immortalised cell line BeWo choriocarcinoma (BeWo) were cultured in DMEM supplemented with 10% foetal bovine serum, penicillin–streptomycin and l-glutamine, at 5% CO_2_ at 37°C. Cells were transiently transfected using Lipofectamine 2000 transfection reagent (Invitrogen) via the manufacturers’ instructions. Cells were transfected in 24-well plates, at a confluency of 80%, with each of four *LHB* promoter inserts, alone or in combination with concentrations of pCMV-myc-ELF5 (0–150 ng), with DNA input controlled with an empty control pCMV-myc vector. Renilla was co-transfected as an internal control at a concentration of 0.05 ng per well. Twenty-four post-transfection, cell lysates were harvested and promoter activity was measured using the Dual-luciferase Reporter Assay (Promega), as per the manufacturer’s instructions.

### Culture of equine trophoblast and scratch assays

Chorionic girdles from day 34 conceptuses were dissected along the junction with the adjacent allantochorion, and chorion and girdle cells were gently scraped from the basement membrane and underlying mesodermal cell layer using a sterile scalpel blade and pure trophoblast cultured as previously described (Cabrera-Sharp *et al.* 2014). Cells used in the study were between passage 3 and 5. Scratch assays were performed as previously described (Liang *et al.* 2007). Confluent monolayers of cells were scratched, both horizontally and vertically, to form a cross shaped wound. The same position on each arm of the wound was imaged at both time 0 and 24 h and the distance moved calculated by subtracting the width of the scratch at 24 h from that at time 0 (following a preliminary experiment performed at 0, 8, 24 and 48 h). Average width of a scratch was calculated by measuring its area and dividing by its length. At time 0 scratches were treated with either vehicle (ethanol used to resuspend the Rhosin) or Rhosin (Insight Biotech), 50 µM, a concentration that has been shown previously to inhibit Rho activation by approximately 80% (Shang *et al.* 2013). Following treatment cells showed no overt changes in morphology or cell death, maintaining a healthy monolayer appearance in all regions outside of the scratch. All experiments were done in duplicate and consisted of four independent wells per treatment, each well measured at four separate points, giving a total of 16 measurements per treatment group per experiment. Statistical differences in cell movement were compared using a paired *t*-test in GraphPad Prism.

## Results

### Transcriptomic profile of chorionic girdle between days 27 and 34 of pregnancy

The data reported below used sibling conceptuses obtained at days 27, 30, 31 and 34 of pregnancy over two breeding seasons (total *n* = 38 samples, *n* = 4–5 each time point and each tissue). All conceptuses used in the study showed normal growth patterns and had normal placental and foetal anatomy as assessed clinically by transrectal ultrasonography and grossly under a dissecting microscope. For mare stallion pairs that did not generate a full set of conceptuses, half-sibling conceptuses were used (Supplementary Table 1).

**Figure 1:**
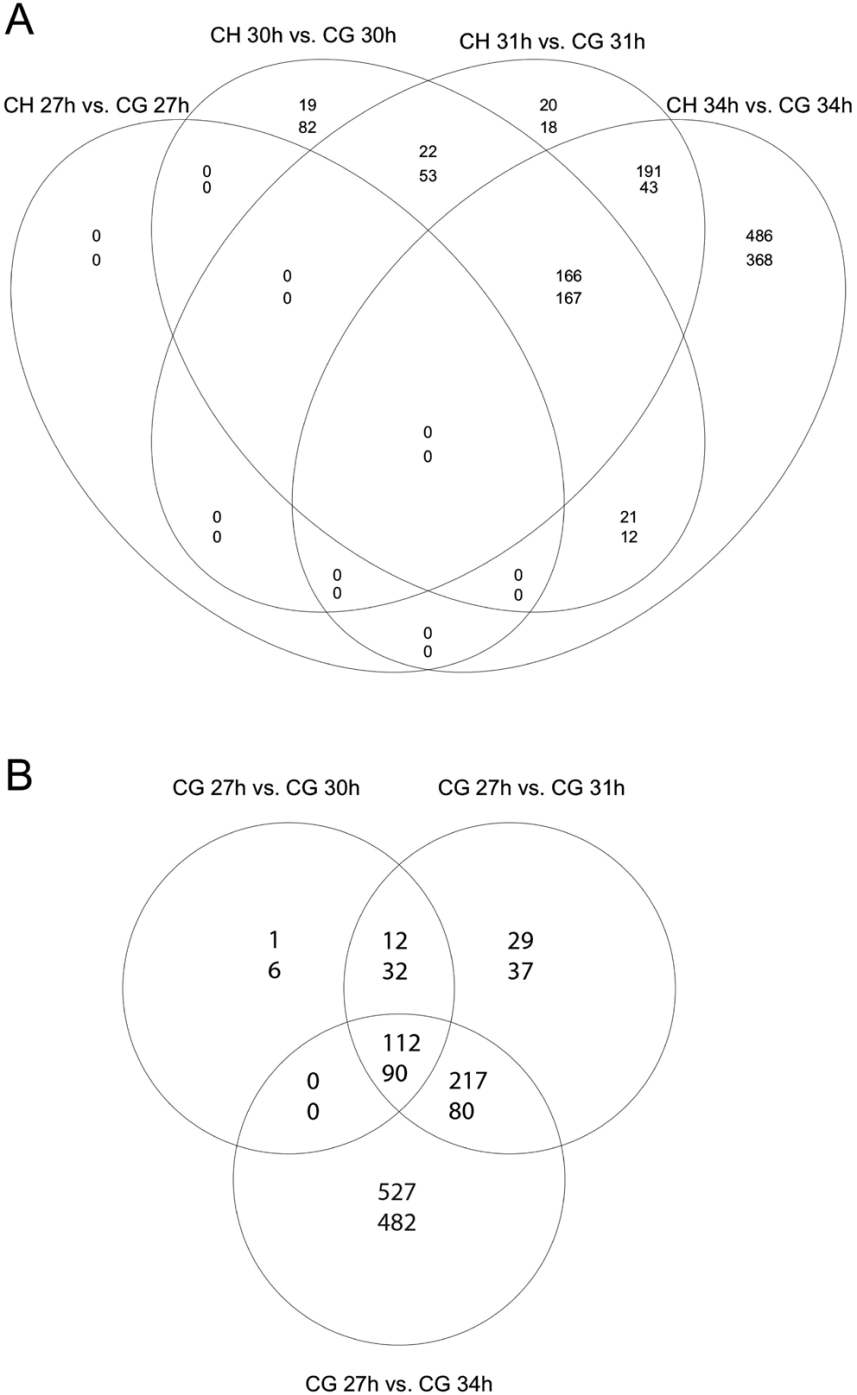
Global changes in gene expression in the chorionic girdle (CG) and adjacent chorion (CH) between days 27 and 34 of pregnancy. Venn diagrams showing the number of up (top number) and down (bottom number) regulated genes that changed in expression in the chorionic girdle compared day 27 chorionic girdle (A) and to the adjacent chorion (B).

The samples grouped well by tissue and gestational age (Supplementary Fig. 2). Analysis of global changes in gene expression revealed that 2207 probes were differentially expressed in the chorionic girdle and chorion across one or more time points or between tissues (Absolute log fold change >2, FDR-adjusted *P* < 0.05) (Supplementary Table 3). Gene expression in the control tissue (chorion) was remarkably stable throughout the gestation period studied, with only 11 genes differentially expressed in the chorion between days 27 and 34 (Table 1). In contrast, gene expression was rapidly induced in the chorionic girdle over the same time period (Table 1), with a peak of 781 genes differentially expressed in the chorionic girdle between day 27 and day 34. Consistent with a wave of genes that are turned on or on and off again during chorionic girdle development, there was significant overlap in the differential expression of genes at day 30, 31 and 34 (Fig. 1A). Similarly, there was a significant increase in differential gene expression between the chorionic girdle and adjacent chorion between days 30 and 34 (Fig. 1B and Table 1). At day 27, no genes were differentially expressed between chorionic girdle and chorion but this rapidly changed during chorionic girdle development and by day 34 there were 1454 probes representing 754 genes that were differentially regulated between the two tissues.

**Table 1:** Number of probes and genes differentially expressed between tissues (CH, chorion; CG, chorionic girdle) and gestation day (days 27–34).

Comparison	Upregulated probes	Upregulated genes	Downregulated probes	Downregulated genes
CH_27 vs CG_27	0	0	0	0
CH_27 vs CH_30	0	0	0	0
CH_27 vs CH_31	0	0	0	0
CH_27 vs CH_34	2	2	17	9
CG_27 vs CG_30	125	60	128	56
CG_27 vs CG_31	370	198	239	119
CG_27 vs CG_34	856	447	652	334
CH_30 vs CG_30	228	123	314	167
CH_30 vs CH_31	0	0	0	0
CH_30 vs CH_34	0	0	0	0
CG_30 vs CG_31	0	0	0	0
CG_30 vs CG_34	277	160	384	207
CH_31 vs CG_31	399	207	281	148
CH_31 vs CH_34	0	0	0	0
CG_31 vs CG_34	70	40	196	101
CH_34 vs CG_34	864	442	590	312

### Validation of genes by qRT-PCR

**Figure 2:**
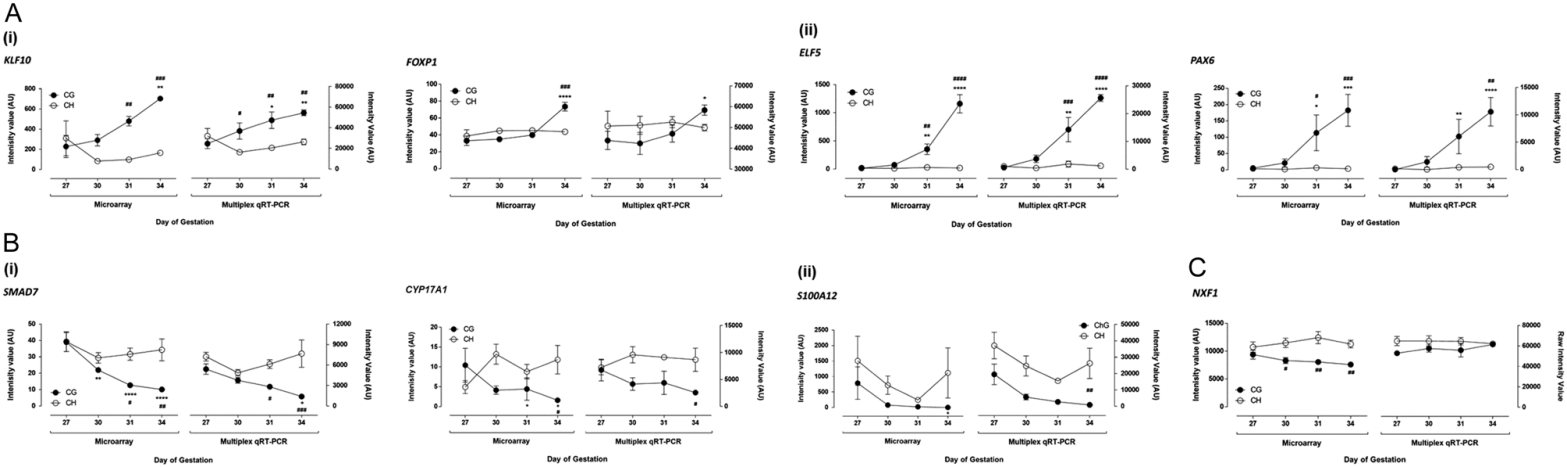
Validation of microarray data using multiplex qRT-PCR. 8 genes selected from microarray data were validated in expression via multiplex qRT-PCR. Microarray data is plotted on the left hand axes, alongside multiplex qRT-PCR data plotted on the right hand axes of each pair of graphs. All fold changes are shown compared with day 27 chorionic girdle. (A) Genes selected for validation with an upregulation (i) 2–5 fold and (ii) >20 fold, in the chorionic girdle between days 27 and 34 of pregnancy in microarray data. (B) Genes selected for validation with a downregulation (i) 2–5 fold and (ii) >20 fold, in the chorionic girdle between days 27 and 34 of pregnancy in microarray data. (C) Gene selected with no change in the chorionic girdle between days 27 and 34 of pregnancy, based on microarray data. *P < 0.05, **P < 0.01, ***P < 0.001, ****P < 0.0001 (compared to day 27 chorionic girdle), ^#^*P* < 0.05, ^##^*P* < 0.01, ^###^*P* < 0.001, ^####^*P* < 0.0001 (compared to chorion at same time point) (two-way ANOVA).

To validate the microarray data, multiplex qRT-PCR was performed. Eight genes were selected from the array to fit the following criteria: up/downregulated with an absolute fold change between 2 and 5 or >20 in the chorionic girdle between day 27 and day 34. Of the eight genes analysed, six of the expression profiles (kruppel like factor 10 (*KLF10*), E74-like factor 5 (*ELF5*), paired box protein 6 (*PAX6*), SMAD family member 7 (*SMAD7*), Cytochrome P450 17A1 (*CYP17A1*) and *NXF1*) were highly reproducible between the microarray data and multiplex qRT-PCR data (Fig. 2). The remaining two genes (S100 Calcium-Binding Protein A12 (*S100A12*) and Forkhead Box Protein 1 (*FOXP1*)) showed very similar patterns of expression in the two tissues, although statistical significance did not always match. The magnitude of the fold change in expression in the chorionic girdle between days differed between assays for some genes, although always remained in the same direction and, with the exception of *FOXP1*, also continued to fall into the same magnitude grouping used for its selection (2–5 or >20). The control gene, *NXF1* did not change in expression in the chorionic girdle at any time point between days 27 and 34. Although the fold change in expression was significantly different between the chorionic girdle and chorion for *NXF1*, the magnitude was always less than 1.5-fold.

### Top upregulated and downregulated gene lists

The 20 top genes that were up/downregulated in the chorionic girdle between day 27 and 34 (absolute fold change >2, *P* < 0.05) are listed in Table 2 (up) and Table 3 (down). Of the top 20 upregulated genes at day 30, 15 genes also appeared in the top upregulated gene list for days 31 and/or 34. *CGA*, *CGB*, *GCM1*, *MHC Class I*, were all in the top 20 upregulated gene list. Interleukin 22, previously shown to be highly upregulated in day 34 chorionic girdle, was not one of the top 20 upregulated genes but was upregulated in day 34 chorionic girdle compared with day 27 chorionic girdle (log fold change 3.94, *P* = 0.001). Top upregulated genes included fatty acid-binding protein 4 (FABP4) that peaked in expression at day 31 and transcription factor BCL6, highly upregulated at days 30 through 34. Immune genes, such as CXCL14, also featured in the top 20 list with expression highly induced as early as day 30.

**Table 2:** Top 20 upregulated genes in the chorionic girdle at day 30, 31 and 34 all compared to day 27 chorionic girdle.

Gene symbol^^^	Gene function	LogFC day 30	LogFC day 31	LogFC day 34
FABP4	Fatty acid binding protein	5.81310***	7.12020****	5.48164***
CG	Glycoprotein hormone alpha subunit	5.24752**	6.85655***	8.54458****
Unamed^a^	Unknown	4.62192**		
BCL6	Transcription factor	4.28718***	5.05434****	5.70755****
SBSPON	Scavenger receptor activity	4.14721**	5.04306***	5.67806****
ENSECAbib17968	Uncharacterised protein	3.61386**	4.59892****	
FFAR2	G-protein-coupled receptor	3.36892*	5.03815***	6.84478****
FGF7	Epithelial cell-specific growth factor	3.35574***	4.99731****	
Unamed^b^	Unknown	3.27125**	4.00893****	4.90052****
PSMB9	Proteasome subunit	3.21874**	4.40427****	5.09380****
ENSECAbib15366	Uncharacterised protein	2.91600***	3.80865****	
PLAC9	Placenta specific gene	2.88399**	4.03647****	
CXCL14	Cytokine	2.82875**		
CCNB1	Regulatory protein involved in mitosis	2.77803**		
ARG2	Arginase activity	2.76454***		
MYBL2	Transcription factor	2.66493**		
RRM2	Ribonucleotide reductase	2.64996**	3.27003***	
Unamed^c^	Unknown	2.58010*	3.43442***	5.48339****
SERPINE2	Inhibitor of serine protease	2.52432*	3.32343**	5.53857****
GCM1	Transcription factor	2.44777*	3.51574***	
Unamed^d^	Unknown		4.93379***	
Unamed^e^	Unknown		3.51056***	5.97793****
FAM227B	Transcription factor		3.31251****	
MHC Class I	Central role in the immune system		3.23287***	
TAF4B	Selective coactivator of transcription		3.20532****	
TCN1	Vitamin B-12 binding protein			8.97029****
LAMB3	Basement membrane protein			7.03224****
Unamed^f^	Unknown			6.30324****
ENSECAbib1317	Known protein coding			6.09937****
Unamed^g^	Unknown			5.95520****
ITGA2	Transmembrane receptor subunit			5.79772****
CADM1	Cell adhesion molecule			5.30317****
Unamed^h^	Unknown			4.92632****
Unamed^i^	Unknown			4.89318****
CGB	Chorionic gonadotrophin B-subunit			6.52371****

Of the top 20 upregulated genes at day 31, 15 also appeared on the list for day 30. Of the top 20 genes upregulated at day 34, 10 genes appeared on the lists for day 30 or 31.

*P < 0.05; **P < 0.01; ***P < 0.001; ^^^unamed gene with Ensembl number; ^a^Equine placenta cDNA Library *Equus caballus* cDNA clone HL02016B1B10, DN509596; ^b^Equine Articular Cartilage cDNA Library *Equus caballus* cDNA clone bib20024A10D12, CX599557; ^c^Equine placenta cDNA Library *Equus caballus* cDNA clone HL02019B2G06, mRNA sequence, DN510553; ^d^Equine placenta cDNA Library *Equus caballus* cDNA clone HL02016B1B10, mRNA sequence (DN509596); ^e^Equine placenta cDNA Library *Equus caballus* cDNA clone HL020001000_PLATE_E04_29_023, mRNA sequence (DN507748); ^f^Equine Articular Cartilage cDNA Library *Equus caballus* cDNA clone bib20021B10E08, mRNA sequence (CX598679); ^g^*Equus caballus* contig07293.EqcaPBMC mRNA sequence (JL622886); ^h^*Equus caballus* contig05471.EqcaPBMC mRNA sequence (JL621232); ^i^Equine placenta cDNA Library *Equus caballus* cDNA clone HL02014A2G04, mRNA sequence (DN508969).

**Table 3:** Top 20 downregulated genes in the chorionic girdle at day 30, 31 and 34 all compared to day 27 chorionic girdle.

Gene symbol^^^	Gene function	LogFC day 30	LogFC day 31	LogFC day 34
FABP1	Fatty acid binding protein	3.42092****		
GPR162	G protein-coupled receptor	3.41190****		
PTGS2	Enzyme for prostaglandin synthesis	3.33766****	3.98743***	4.98492****
RDH16L	Enzyme, retinol dehydrogenase	3.26851****	4.19061****	4.88511****
IL1B	Cytokine	3.25615****	4.55809**	4.92065**
Unamed^a^	Unknown	3.19614****	5.10370****	6.14734****
Unamed^b^	Unknown	3.16932****	4.18707****	4.69047****
ENSECAbib22537	Uncharacterised protein	3.15012****	4.21925****	5.01506****
ENSECAbib7031	Uncharacterised protein	3.00171****		
ACTG2	Cytoskeletal protein	3.00165****		
FMO1	Oxioreductase	2.89347**	3.89842****	4.87058****
CA7	Reversible hydration of CO_2_	2.83819****	3.84538****	
HSD3B1	Bifunctional enzyme	2.75848****		
ENSECAbib19764	Uncharacterised protein	2.63330****	3.64952****	
APL1	membrane-associated glycoprotein	2.61260****	4.56274****	4.66107****
ENSECAbib20332	Uncharacterised protein	2.54894****		
LGMN	Enzyme, cysteine protease	2.52946****		
MT	Golgi localised protein	2.51741****		
ENSECAbib21165	Uncharacterised protein	2.50448****	3.54138****	
CYP17A1	Enzyme, cytokine p450	2.50254****	3.70293****	4.89858****
Unamed^c^	Unknown		4.11917****	
TAGLN3	Actin filament binding		4.11782****	5.72992****
LHFPL1	Transmembrane protein		4.01210***	5.24291****
ENSECAbib24713	Uncharacterised protein		3.74453***	5.23593****
CPVL	Carboxypeptidase		3.73216***	5.52317****
PLXNB1	Regulation of actin cytoskeleton		3.64882****	
ENSECAbib16216	Uncharacterised protein		3.64030****	4.73441****
ENSECAbib6927	Uncharacterised protein			5.32239****
SLC46A1	Transmembrane folate transporter			5.05205****
Unamed^d^	Unknown			5.02691****
Unamed^e^	Unknown			4.78917****
CA4	Reversible hydration of CO_2_			4.69078****
PLA2G10	Enzyme, phospholipase			4.57344****

Of the top 20 upregulated genes at day 31, 16 also appeared on the list for day 30. Of the top 20 genes upregulated at day 34, 10 genes appeared on the lists for day 30 or 31.

*P < 0.05; **P < 0.01; ***P < 0.001; ^^^Ensembl number listed for unnamed genes; ^^^Unamed gene with Ensembl number; ^a^Equine placenta cDNA Library *Equus caballus* cDNA clone HL020010000_PLATE_G06_47_050, mRNA sequence (DN508408); ^b^Equine placenta cDNA Library *Equus caballus* cDNA clone HL02021A2F04, mRNA sequence (DN510935); ^c^Equine placenta cDNA Library *Equus caballus* cDNA clone HL02019B1D12, mRNA sequence (DN510457); ^d^Equine placenta cDNA Library *Equus caballus* cDNA clone HL020002000_PLATE_G01_7_004, mRNA sequence (DN507842); ^e^Equine Articular Cartilage cDNA Library *Equus caballus* cDNA clone bib2041A1D02, mRNA sequence (CX605310).

### Clustering of genes into expression patterns

**Figure 3:**
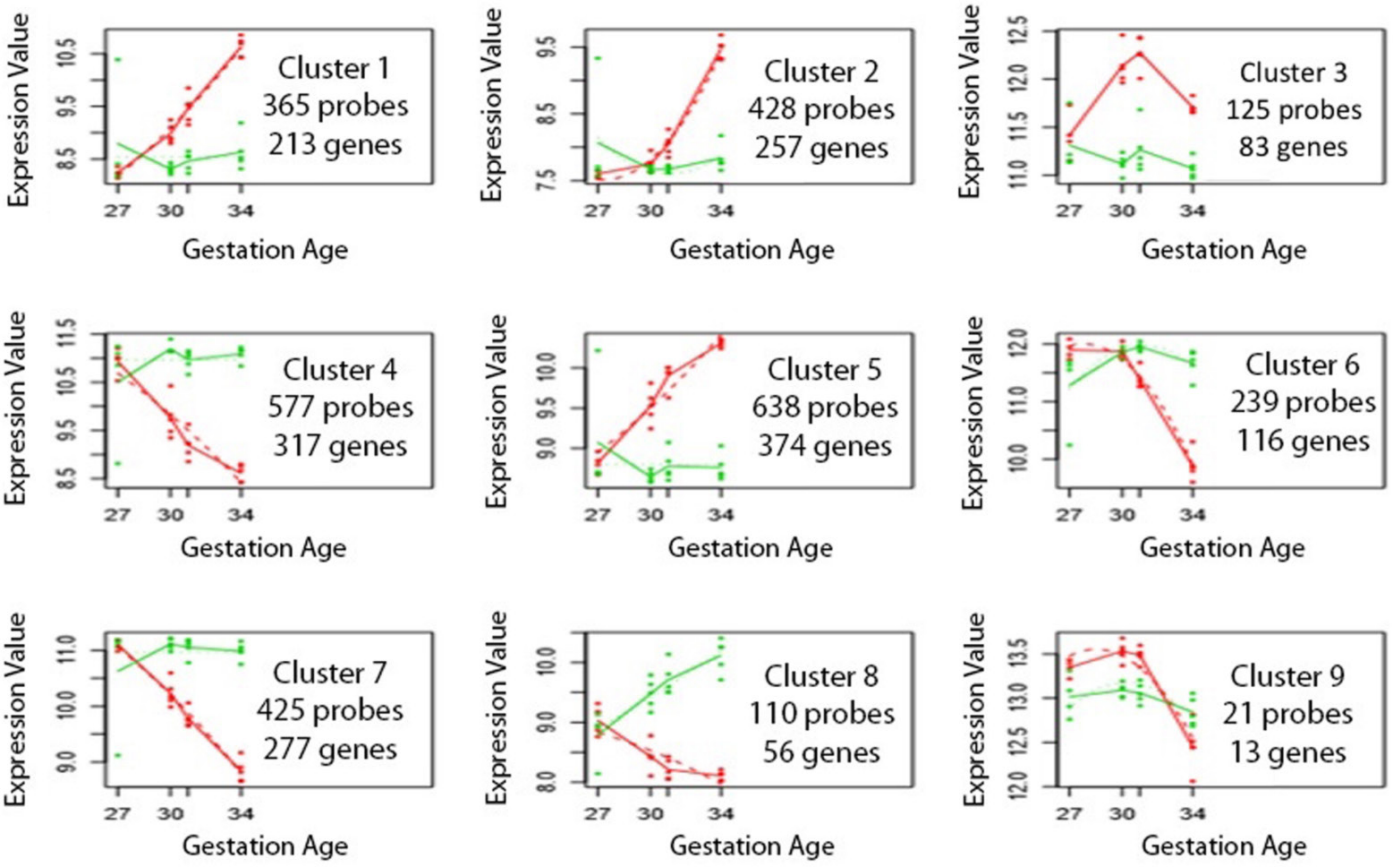
Clusters of similarly regulated genes in the chorionic girdle, identified using maSigPro software. Genes with a fold change >2 (*P* < 0.05) between days 27 and 34 of gestation in the chorionic girdle, were grouped by maSigPro software into the nine most likely expression profiles. Chorionic girdle is shown in red and chorion in green. Plotted is the median expression profile of genes within each cluster. Probe numbers and corresponding gene numbers (including those unnamed genes) are provided below each cluster. Gene lists for each cluster can be found in Supplementary Table 4.

Next, the R package SigPro software was used to cluster genes based on their expression patterns across both time and tissues. Nine different gene expression profiles were identified (Fig. 3) each containing between 13 and 374 genes with an R2 regression fit of >0.7. Supplementary Table 4 provides full gene lists for each cluster. For example, genes included in the gene list for cluster 1 increased in expression in the chorionic girdle (Fig. 3, cluster 1, red) and remained constant in expression in the chorion (green) over the same time period. Genes in clusters 1 and 7 which remained relatively constant in expression in the chorion and gradually increased or decreased in expression in the chorionic girdle between days 27 and 34 (cluster 1 and 7) were identified as genes and components of signalling pathways likely to be involved in cellular growth as either regulators of proliferation and/or differentiation or as markers of the change in the cells differentiation state. Gene lists from these two clusters were input into DAVID functional annotation tool, which identified 37 pathways (*P* < 0.01) (Table 4). Pathways of note were epidermal growth factor (EGF) and vascular endothelial growth factor (VEGF) signalling (11 and 10 entities respectively), integrin signalling (22 entities) and cytoskeletal regulation by Rho GTPases (10 entities). Genes that showed a similar pattern to clusters 1 and 7 but showed higher expression values by day 30/31 were clustered into groups 4 and 5. Four pathways were enriched in this gene list including genes related to the function of the lysosome (13 genes) and cytoskeletal regulation by Rho GTPases (9 entities). Genes that remained relatively constant in the chorion and rapidly increased in expression in the chorionic girdle at day 34 more consistent with genes associated with the acquisition of an invasive phenotype were also identified (Fig. 3 and Supplementary Table 3, clusters 2 and 6). The pathways represented by clusters 2 and 6 included focal adhesion (16 entities), chemokine signalling (11 entities) and tight junctions (10 entities) (Table 4). Cluster 3 revealed 83 genes that peak at days 30/31 specific to when differentiation is initiated (Supplementary Table 3, cluster 3 gene list). Clusters 3, 8 and 9 were not further analysed in pathway analysis due to relatively low total number of genes on these lists.

**Table 4:** Pathways and processes enriched in gene clusters.

Term	No. of genes	*P* value
Cluster 3		
Spliceosome	5	0.00016
Clusters 4 and 5		
Lysosome	13	0.00110
Cytoskeletal regulation by Rho GTPase	9	0.00610
Pathogenic *Escherichia coli* infection	8	0.00230
Ether lipid metabolism	6	0.00290
Clusters 1 and 7		
Pathways in cancer	22	0.00002
Integrin signalling pathway	22	0.00000
Inflammation mediated by chemokine and cytokine signalling pathway	21	0.00007
Focal adhesion	19	0.00000
Regulation of actin cytoskeleton	17	0.00003
Angiogenesis	14	0.00320
PDGF signalling pathway	13	0.00140
Chemokine signalling pathway	12	0.00260
Neurotrophin signalling pathway	12	0.00006
EGF signalling pathway	11	0.00180
Cytoskeletal regulation by Rho GTPase	10	0.00083
VEGF signalling pathway	10	0.00005
Small cell lung cancer	10	0.00004
Renal cell carcinoma	10	0.00001
Tight junction	9	0.00660
Endothelin signalling pathway	9	0.00110
Prostate cancer	9	0.00038
Chronic myeloid leukaemia	9	0.00010
T cell receptor signalling pathway	8	0.00580
Fc gamma R-mediated phagocytosis	8	0.00260
Insulin signalling pathway	8	0.00210
ErbB signalling pathway	7	0.00620
Progesterone mediated oocyte maturation	7	0.00590
ECM-receptor interaction	7	0.00510
Colorectal cancer	7	0.00510
Fc epsilon RI signalling pathway	7	0.00340
Pancreatic cancer	7	0.00210
Melanoma	7	0.00200
Glioma	7	0.00097
Acute myeloid leukaemia	7	0.00059
Endometrial cancer	7	0.00030
Valine, leucine and isoleucine degradation	7	0.00010
p53 pathway feedback loops 2	6	0.00750
Non-small cell lung cancer	6	0.00230
Bladder cancer	6	0.00059
Glycerolipid metabolism	5	0.00530
Thyroid cancer	5	0.00071
Clusters 2 and 6		
Focal adhesion	16	0.00001
Chemokine signalling pathway	11	0.00410
Adherens junction	10	0.00001
Tight junction	10	0.00110
Histamine H1 receptor mediated signalling pathway	6	0.00076
Vibrio cholera infection	5	0.00950
GABA-B receptor II signalling	5	0.00350

The genes identified in maSigPro clusters 3, 4 and 5, 1 and 7 and 2 and 6 were mapped onto pathways in the KEGG and PANTHER databases. Pathways containing a minimum of five cluster genes (*P* < 0.01) were considered biologically relevant. Shown are the pathway identifiers, the number of cluster genes present in the pathway and the *P* value (Fishers Exact).

### Top regulator effector networks

Next, IPA was used to further explore the effector networks active during development of the chorionic girdle. Networks in the chorion were not assessed as only 11 genes were differentially expressed in day 34 chorion (compared to day 27) and no genes differed in expression at other time points. The top 5 regulated effector networks at days 30, 31 and 34 (compared with day 27 chorionic girdle) are listed in Table 5. Activation scores were all high, suggesting that gene expression data is highly supportive of the networks identified. Consistent with key cellular processes known to occur during chorionic girdle development, two cellular processes were highly represented in these lists; cell movement/cell migration/invasion (6/15 networks) and proliferation/differentiation (5/15 networks). Cell movement and migration pathways demonstrated an increase in activation score over time from 8.485/6.062 at day 30 to 11.25 at day 31 and scores of 10.8, 9.865 and 8.014 for migratory pathways at day 34. By day 31, cell cycle regulation and S phase checkpoint control (activation score 9.925) were processes induced in trophoblast and this continues to day 34. Based on the directionality of the expression change in the microarray data, a number of upstream regulators of these effector networks were identified and are also listed in Table 5.

**Table 5:** Top 5 effector networks and associated upstream regulators identified using IPA.

Day of gestation	Top upregulated upstream regulators (vs D27 ChG)	Functions of regulated effector networks	Consistency score
D30	1. GLIS2, IgG, IHH,MAP2K1, MAP2K1/2, MAP2K3, miR141-3p, PTHLH	Cell movement of tumour cells	8.485
	2. Akt, CTNNB1, ERK, ETV4, Histone H3, PRKCZ	Failure of kidney, glomerulosclerosis	7.778
	3. ETV4, PML, SDCBP, TBX3, TFEB	Accumulation of lipid, cell movement of tumour cells, blood vessel development	6.306
	4. ETV4, SP11, TLR4	Cell movement of tumour cells, fibrosis of kidney, urinary system morphology	6.062
	5. E2f, EP400, LYL1, RAF1, TBX2, TFDP1	Size of animal, size of embryo	5.277
D31	1. BGN, ETV4, ETV5, GLIS1, HNRNPAB, MAP2K1/2, PLCD1, PTHLH, SOCS1, SRC (family)	Migration of cancer cells	11.250
	2. AHR, BMIP3L, CCND1, E2F2, E2F6, EIF4G1, let-7, PPM1D, RBL1, STUB1, TBX2	Repair of DNA, S phase checkpoint control	9.922
	3. E2f, EP400, Pka, TBX2, TFDP1	Proliferation of heart cells, size of embryo, quantity of brain cells	4.750
	4. CDKN1A, E2F1, HDAC1, let-7, mir-10, miR16-5p, Rb, TBX2	S phase checkpoint control	4.596
	5. HNRNPAB, MAP3KB, NOS2, POU5F1, SATB1, SPDEF	Progression of tumour	3.273
D34	1. APC, ETV4, ETV5, GLIS2, MAP3K8, PLCD1, SPARC, WISP2	Cell movement of lymphocytes, migration of cancer cells	10.800
	2. BMP15, BMPR1A, HNRNPAB, PLA2G5, TBX5	Angiogenesis, cell movement of lymphocytes, colony formation of cells movement of cancer cells, proliferation of muscle cells	9.865
	3. GLIS2, MAP2K1/2, PLCD1, SMAD3, SRC family, TGFB3, WISP2	Migration of tumour cells	8.014
	4. CCND1, E2F2, E2F6, EIF4G1, let-7, PPM1D, RBL1, STUB1, TBX2	Repair of DNA, S phase checkpoint control	7.649
	5. Akt, CDKN1A, E2F1, FGF2, HDAC1, let-7, mir-10, miR125b-5p, miR16-5p, PTEN, Rb, TBX2	S phase checkpoint control	5.303

The top 5 upregulated regulator effect networks at each time point, as compared to day 27 chorionic girdle are shown. Identified upstream regulators and their downstream cellular effects are shown, with consistency score for the pathway (*z*-score >2, *P* < 0.05, IPA).

### Top canonical pathways

**Figure 4:**
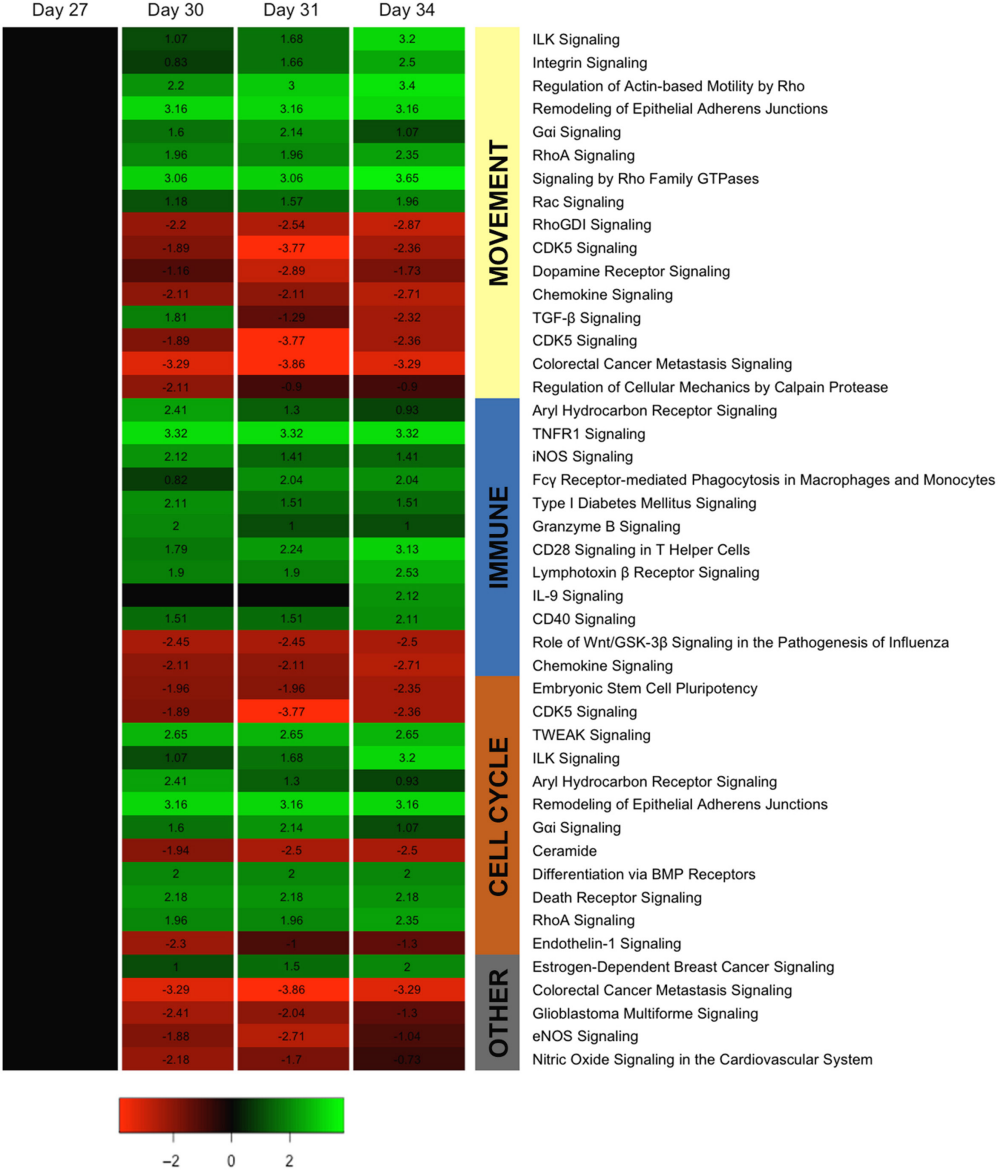
Activation of canonical pathways in the chorionic girdle during day 27–34 of pregnancy. IPA was used to identify activation or repression of canonical pathways in the chorionic girdle between days 27 and 34 of gestation. All pathways identified by the programme to have an activation (*z*-score) score of > or <2 and *P* < 0.05 are shown with green indicating activation and red inactivation. Pathways were grouped according to known functions.

IPA was also used to assess the activation of canonical pathways in the chorionic girdle between days 27 and 34 of pregnancy using the full gene list. There were dynamic changes in predicted pathway activation in the chorionic girdle during development (Fig. 4). Out of a total of 533 pathways stored in the IPA software at the time of analysis, 35 pathways had an activation *z*-score of >2 or <−2 (*P* < 0.05) at one or more time points during chorionic girdle development (Fig. 4). These included 16 pathways known to be associated with cell movement, 12 pathways known to be involved in cellular immune responses and 12 pathways associated with cell cycle regulation and/or differentiation (Fig. 4). There was a predominance of movement pathways related to signalling by Rho GTPases including activation of RhoA, regulation of actin-based motility by Rho and inhibition of the inhibitory signal, RhoGDI signalling (Fig. 4). Cell cycle pathways with positive activation scores included ILK signalling, aryl hydrocarbon receptor signalling (which peaked at day 30 around initiation of differentiation) and TWEAK signalling.

### Validation of genes and pathways

Next, in order to further explore the activity of the genes and pathways identified above, one gene and two pathways were selected for further investigation.

### ELF5 transactivates the CGB promoter

**Figure 5:**
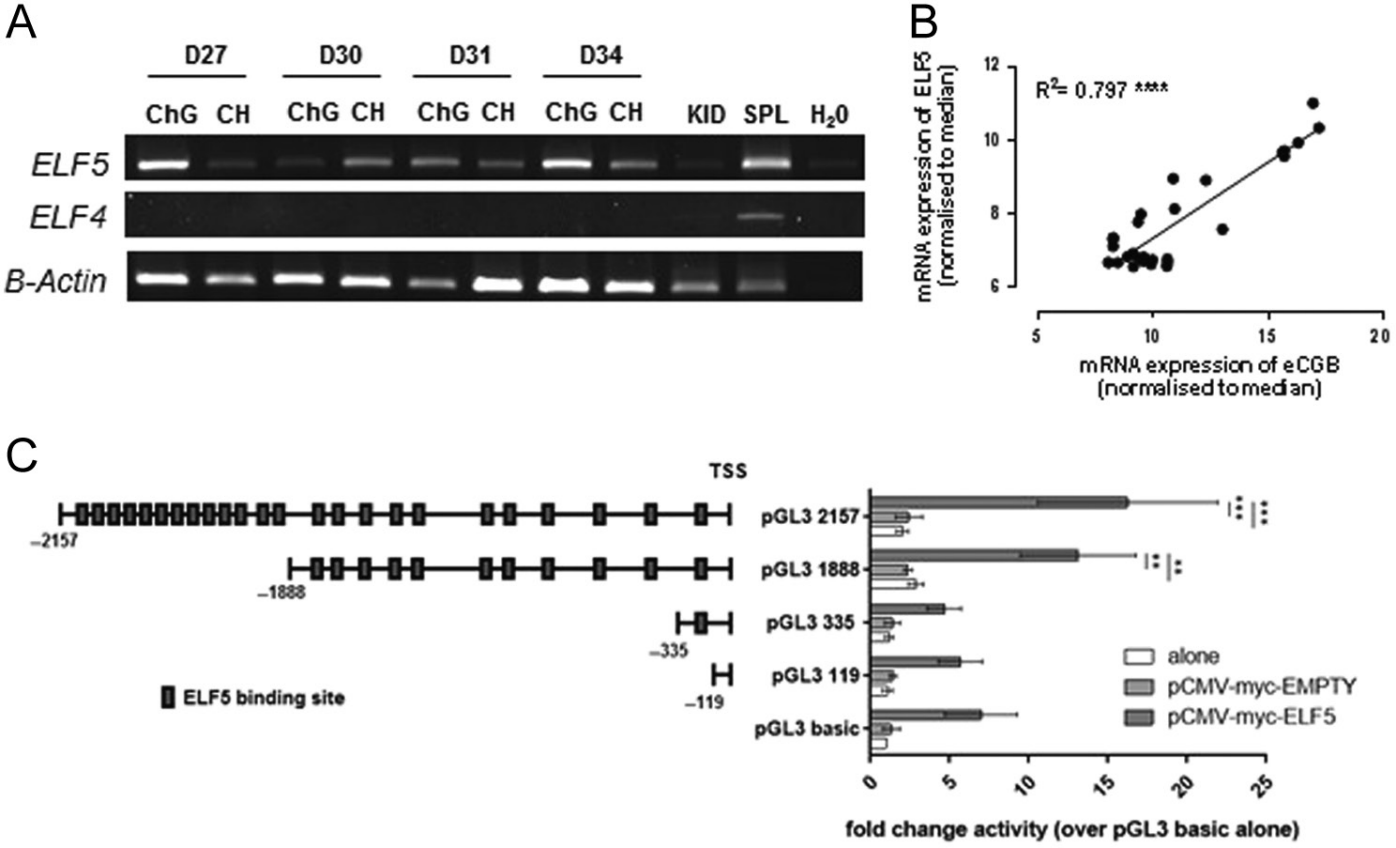
*ELF5* mRNA is expressed in chorionic girdle trophoblast cells, correlates with *CG/LHB* mRNA expression and drives CG/LHB promoter activity. (A) *ELF4* and* ELF5* mRNA in equine chorionic girdle and control chorion tissues at days 27, 30, 31 and 34 of gestation as shown by RT-PCR with B-actin used as a loading control. Kidney and spleen tissues are used as control. (B) Correlation between *ELF5* and *CGB* mRNA expression in individual day 30–34 equine chorionic girdle and chorion samples. Microarray expression data is normalised to the median in R software and displayed as fold change. (C) Truncated promoter inserts, as shown with annotated potential ELF5 binding sites, were transfected into COS7 cells alone, in the presence of 150 ng pCMV-myc-Empty or 150 ng pCMV-myc-ELF5. Numbers are relative to Translation Start Site. Promoter activity is expressed as fold change activity over pGL3-basic alone. **P < 0.01, ***P < 0.001. (*n* = 3, two-way ANOVA) activity is expressed as fold change activity. Over pGL3-basic alone. **P < 0.01, ***P < 0.001. (*n* = 3, two-way ANOVA).

Ets family transcription factor ELF5 was selected from the genes confirmed by qRT-PCR for further investigation due to its temporal expression profile that suggested a possible role in regulation of trophoblast development. RT-PCR amplification of ELF5 and ELF4 (a related family member) genes in equine chorionic girdle and control chorion tissues from gestational ages 27 to 34 days (Fig. 5A) showed that ELF5 was expressed in the chorionic girdle and chorion at all time points, with evidently higher expression it the chorionic girdle compared to the chorion. Gene expression of ELF4 was not detectable in any equine conceptus tissues tested, regardless of gestational age. Expression in control spleen tissue demonstrated successful amplification of the ELF4 gene (Fig. 5A). Western blot analysis confirmed the presence of an approximately 37 kDa protein in the chorionic girdle similar to the predicted size of ELF5 (31 kDa) (Supplementary Fig. 3).

This expression profile in the chorionic girdle combined with bioinformatics analysis of the CG/LH promoter that revealed 24 predicted ELF5-binding sites (data not shown) led us to hypothesise that ELF5 may play a role in inducing CG/LHB expression, a key gene induced during differentiation of chorionic girdle cells and the rate-limiting subunit required for eCG production (de Mestre *et al.* 2009). To explore this further, we used the expression data generated on the microarray to determine if an association existed between CG/LHB and ELF5 expression in all chorion and chorionic tissues (Fig. 5B). ELF5 and CG/LHB mRNA expression in individual chorionic girdle and chorion tissues was strongly positively correlated (*R* ^2^ = 0.80, *P* < 0.0001).

To determine whether activity of the CG/LHB promoter was driven by ELF5, co-transfections were carried out in COS7 cells using 250 ng of four truncated *CG/LHB* promoter inserts (Read *et al.* 2018), either alone, with addition of 150 ng pCMV-myc-Empty vector, or with 150 ng pCMV-myc-ELF5 (Supplementary Fig. 4). Promoter activity was assessed using luciferase assays (Fig. 5C). The 2157 bp *CG/LHB* construct that contained 24 predicted binding sites for ELF5 was transactivated by pCMV-myc-ELF5, 8.4-fold (s.e.m. = ±4.4, *P* = 0.0002) over pCMV-myc-Empty. The 1888 bp *CG/LHB* promoter, containing 11 predicted binding sites was driven 5.8-fold (s.e.m. = ±2 *P* = 0.0023) by pCMV-myc-ELF5 above pCMV-myc-Empty. No significant increase in promoter activity was observed for the pGL3-basic, pGL3-119 (no predicted sites) or pGL3-335 (1 predicted site) promoter constructs when co-transfected with pCMV-myc-ELF5. The pCMV-myc-Empty vector had no driving activity on any of the promoter constructs.

### Validation of classical MAPK signalling in the chorionic girdle

**Figure 6:**
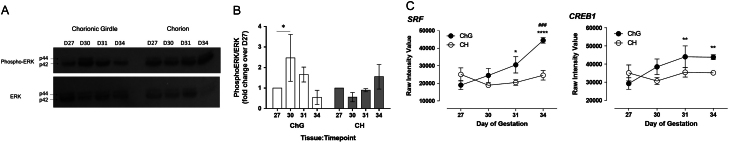
Activity of the ERK1/2 pathway in the chorionic girdle. (A) Representative western (*n* = 3) for phospho-ERK and total ERK in one timecourse set of chorionic girdle and chorion tissues. Shown are the phosphorylated and total p44 (ERK2) and p42 (ERK1) protein. (B) Densitometry analysis of western blots for phospho/Total ERK (P44/42) *n* = 3. *P < 0.05. (C) Multiplex qRT-PCR was used to quantify mRNA expression of SRF and CREB1. *P < 0.05, **P < 0.01, ****P < 0.0001 (compared to day 27 chorionic girdle), ^###^*P* < 0.001 (compared to chorion at same timepoint) (two-way ANOVA).

Signalling molecules downstream of a number of pathways predicted above to be modulated during chorionic girdle development (FGF, EGF and VEGF) included classical mitogen-activated protein kinase (MAPK) signalling molecules, ERK1/2. Western blot analysis demonstrated that ERK/MAPK pathways were active in the both the chorionic girdle and chorion at all time points as demonstrated by detectable p44/p42 protein bands (Fig. 6A). An increase in phosphorylation of the p44 (ERK2) protein at day 30 of pregnancy in the chorionic girdle was observed with no apparent change in the phosphorylation of the p42 protein (ERK1). Densitometry analysis of phospho ERK1/2/Total ERK1/2 (*n* = 3 conceptuses per time point) showed a significant increase in phospho-ERK at day 30 compared with day 27 in chorionic girdle (*P* = 0.0348) but not chorion (Fig. 6B). In addition, using qRT-PCR, we assessed the expression profile of ERK1/2-regulated transcription factors, serum response factor (*SRF*) and CRE-binding protein 1 (*CREB1*) in the chorionic girdle. Correlating with the increase in ERK1/2 activity at day 30, *SRF* and *CREB1* expression significantly increased at day 31 in the chorionic girdle when compared to day 27 (Fig. 6C).

### RhoA is involved in migration of equine chorionic girdle trophoblast

**Figure 7:**
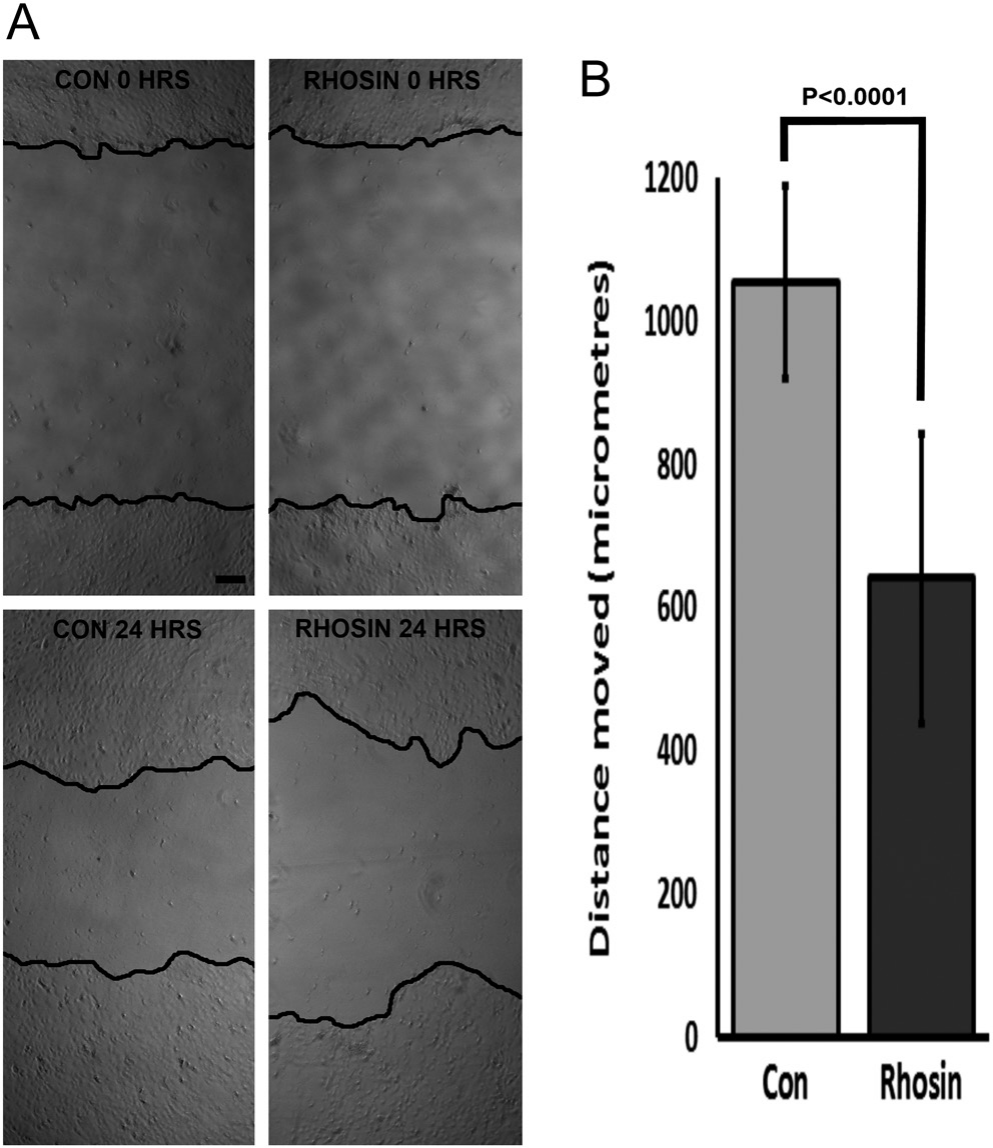
Inhibition of Rho function decreases motility of primary equine trophoblast cells. (A) Representative images of scratches at time 0 and 24 h in the absence and presence of Rho inhibitor (Rhosin, 50 µM). Black lines in each image mark the margins of the scratch and show a decreased motility following Rhosin treatment. (B) Cumulative data of average distance moved showing significantly (*P* < 0.0001) decreased motility in Rhosin treated cultures compared with control. Data shown are mean and standard deviation for 24 measurements (*n* = 3 independent experiments in day 34 chorionic girdle trophoblast). Scale bar in A represents 200 µm.

A number of pathways related to Rho signalling were identified in Fig. 4. In order to further explore the functionality of these pathway, primary day 34 equine trophoblast cells were cultured in the presence or absence of Rho inhibitor Rhosin (50 µM) and trophoblast migration assessed using a scratch assay (Fig. 7A). There was no change in the morphology of the cells or evidence of cell death following treatment with Rhosin or ethanol (control). Treatment of chorionic girdle trophoblast with 50 µM Rhosin resulted in a significant reduction in trophoblast migration into the scratch compared to ethanol control treated cells (*P* < 0.001) (*n* = 8, 4 readings per scratch) (Fig. 7B).

## Discussion

Chorionic girdle development requires a number of co-ordinated processes that must occur in a timely and controlled manner. Establishing the global gene expression profile for genetically paired conceptuses over a 7-day period of pregnancy (day 27–34) has provided a powerful insight into the genetic landscape of the trophoblast cells as they terminally differentiated from uninucleate trophoblast into eCG-secreting binucleate trophoblast with an invasive phenotype. Fluctuations in gene expression were both dynamic and very specific to the CG whilst the adjacent chorion had remarkably stable gene expression over the same time period. The abundant genes and pathways identified were highly consistent across laboratory and bioinformatic approaches, with three of these, ELF5 function, ERK1/2 activity and Rho A function, further confirmed in functional assays.

Initial PCA visualisation of grouping of the tissues demonstrated distinct gene expression profiles in the chorionic girdle at all time points assessed, whilst in contrast, chorion tissues from all time points were genetically identical. In total, only 11 genes were differentially expressed in the control tissue, chorion compared with 1625 in the chorionic girdle. Although chorionic girdle trophoblast at day 30/31 are still primarily uninucleate trophoblast, the gene profile already had substantially shifted (116 and 317 genes respectively) when compared to day 27 cells. This corresponds to the time at which a large proportion of chorionic girdle trophoblasts rapidly proliferate and undergo differentiation to become terminally differentiated binucleate trophoblast cells and is also the period immediately prior to the significant induction of eCG expression (de Mestre *et al.* 2009, Read *et al.* 2018). The genes and pathways switched on/off in this early period in girdle development will provide important clues to the initiators of proliferation, differentiation and eCG expression. For example, fatty acid-binding protein FABP4, a gene known to regulate both proliferation and differentiation genes in human trophoblast cell line HTR8/SVneo (Basak *et al.* 2018), was found to peak in expression at day 31. C-X-C Motif Chemokine Ligand 14 (CXCL14) expressed in human villous cytotrophoblast (Kuang *et al.* 2009) and known to negatively regulate trophoblast invasion was highly induced by day 30. It is plausible that CXCL14 is playing a similar role in the chorionic girdle, preventing premature invasion of early forming binucleate cells into the endometrium, which usually does not take place until day 34–38.

In order to further establish that changes in gene expression observed in microarray analysis were truly representative of the biology of the trophoblast cells eight genes were verified by qRT-PCR and all demonstrated the same expression pattern to that of the microarray. A small number of genes shown previously to be expressed in day 34 chorionic girdle (Bacon *et al.* 2002, de Mestre *et al.* 2009, Brosnahan *et al.* 2012) featured on gene and signalling pathway lists providing further confirmation of the validity of the large dataset. For example, gene cluster analysis identified the SMAD1/5 arm of the TGFβ signalling pathway to contain multiple differentially regulated genes in the chorionic girdle, including *BMP4*, its receptor bone morphogenic protein receptor 2 (*BMPRII*) and *SMAD1/5*, all consistent with a known function of this pathway in equine trophoblast (Cabrera-Sharp *et al.* 2014). EGF signalling in chorionic girdle cells as measured by canonical pathway analysis increased in activity in the chorionic girdle supporting previously reported expression profiles of EGF and EGF receptors in the chorionic girdle (Stewart *et al.* 1994, Allen *et al.* 2017). The function of EGF in the chorionic girdle is not known but in human trophoblast, EGF has been shown to promote proliferation through activation of AKT and ERK1/2 signalling downstream of EGFR (Fock *et al.* 2015, Costa *et al.* 2016). The ERK/MAPK signalling pathway was confirmed here to be functional in chorionic girdle trophoblasts, increasing in activity at day 30 of pregnancy when compared to day 27. It is therefore likely that EGF signalling acting via ERK1/2 is indeed active in the chorionic girdle.

Phosphorylation of p44/p42 and nuclear translocation is thought to be important for cell cycle re-entry into S phase in response to mitogenic activators (Brunet *et al.* 1999). S phase checkpoint effector networks are identified at days 31 and 34 here, so it is possible that the MAPK/ERK signalling pathway is crucial for this regulation. Other described roles for the MAPK/ERK pathway include regulation of cellular growth differentiation (Brunet *et al.* 1999, Dumaz & Marais 2005). Due to the ubiquitous nature of the MAPK/ERK pathway, it is likely that it plays roles in multiple cellular processes during chorionic girdle development, perhaps activating the cell cycle at day 30-31 and later differentiation at day 31-34.

Fibroblast growth factor 7 (*FGF7*), a growth factor expressed by bovine immature trophoblast giant cells (Pfarrer *et al.* 2006), was significantly upregulated between day 27 and 34, as was the related family member fibroblast growth factor-binding protein 1 (*FGFBP1*). Previous studies have reported fibroblast growth factor 2 (FGF2), its receptors (fibroblast growth factor receptor 1-4 (FGFR1-4)) and FGF-binding protein to be expressed in equine trophectoderm earlier in pregnancy between days 14 and 28 (de Ruijter-Villani *et al.* 2013). Supportive of a possible autocrine function for FGF7 in the chorionic girdle, FGF signalling was identified in gene cluster analysis with 9 FGF regulated genes found to be induced/repressed between days 27 and 34 and downstream ERK1/2 activity confirmed in chorionic girdle by western blot. This is consistent with other species, where FGF proteins are thought to play a key role in trophoblast development including hCG production (Jeong *et al.* 2016) via signalling through ERK (Taniguchi *et al*. 2000, Yang *et al.* 2011, Kunath *et al.* 2014). The role for FGF signalling in the equine placenta remains unknown, but data here suggest an autocrine function for FGF signalling in trophoblast development in the chorionic girdle. Based on its high expression pattern, it is plausible that it may also act in a paracrine manner on adjacent tissues such as the allantochorion thus co-ordinating chorionic girdle development and implantation.

*ELF5* mRNA expression was found to be one of the most markedly induced genes between days 27 and 34 with maximal expression at day 34. Transactivation studies demonstrated that ELF5 was able to drive the CG/LHB promoter between the regions of 335 and 2500 bp relative to the translation start codon suggesting it may play a role in biochemical differentiation of trophoblast. The large number of potential ELF5-binding sites in this region suggests that ELF5 may drive promoter activity by acting directly to multiple sites in the distal promoter, although further work is required. ELF5 was considered widely considered to be a ‘gatekeeper’ of stem cell status in mouse studies (Hemberger *et al.* 2010, Pearton *et al.* 2011, 2014). More recently, work suggests that ELF5s function changes in a concentration-dependent manner with high levels of ELF5 triggering trophoblast differentiation (Latos *et al.* 2015) achieved through an interaction with transcription factor AP-2 gamma (TFAP2C). Interestingly, we did detect ELF5 transcript in day 27 chorionic girdle, albeit at much lower levels (data not shown). Therefore, it is plausible that the chorionic girdle trophoblast utilise ELF5 in a similar manner to mouse cells, with low levels that ELF5 playing a role in maintaining the stem cell status of progenitor chorionic girdle cells (supported here in the pathway analysis which show loss of stem cell pathways between day 27 and 34) and high levels of ELF5 at day 34 driving the switch to differentiation and induction of CG/LHB expression.

Proliferation pathways consistently arose in regulator effect networks at all time points and were represented in canonical pathway analysis by pathways, such as endothelin 1 signalling and cyclin dependent kinase 5 (CDK5) signalling. The kinetics of these pathways suggest a reduced, yet ongoing requirement for trophoblast proliferation throughout chorionic girdle development. The upstream regulator EP400 was identified as a top upstream regulator at days 31 and 34 of pregnancy and is known to play a role in cellular proliferation in other cells types (Mattera *et al.* 2009). The majority of other signalling data suggests repression of proliferation signalling pathways. Endothelin signalling in cancer cells is known to play diverse roles in proliferation, apoptosis and migration (Rosano *et al.* 2013) and TNF-related weak inducer of apoptosis (TWEAK) signalling is well defined to promote proliferation of cardiomyocytes (Novoyatleva *et al.* 2010). It is likely that pathway suppression represents a continuous but proportionally decreasing population of proliferating cells, as increasing numbers of chorionic girdle trophoblasts terminally differentiate, losing their proliferative capacity.

A number of pathways associated with cellular differentiation emerged, including those involved in cell cycle regulation, loss of pluripotency and AHR and MAPK/ERK signalling pathways. Repair of DNA and S phase checkpoint control were consistently identified throughout chorionic girdle development as important effector networks. Additionally, transcription factors E2 factor (E2F) and E2 factor 2 (E2F2) were identified as top upstream regulators at all time points in the chorionic girdle. The canonical E2Fs (E2F1, E2F2 and E2F3) regulate trophoblast giant cell differentiation through the promotion of the cell endocycle, via regulation of cyclin E and S phase control (Chen *et al.* 2012, Ouseph *et al.* 2012). A conserved role for E2F proteins in regulation of cyclins D and E and subsequent G1 cell cycle progression has been observed in human placental cell differentiation (Ohtani *et al.* 1995). The data generated here suggest that the role for E2F and E2F2 are also conserved in the regulation of the cell cycle in differentiating equine chorionic girdle trophoblasts. Canonical pathway heatmaps indicated that cyclin signalling pathways were upregulated in the chorionic girdle at day 30 of pregnancy, with a subsequent loss of activation at days 31 and 34. Equine trophoblasts differ from human and mouse counterparts (MacAuley *et al.* 1998), in that they are binucleate as opposed to multinucleate and do not possess the increased gene number observed in endoreduplicated cells (Wooding *et al.* 2001). Nevertheless, it is clear that regulation of cell cycle progression and cyclin signalling pathways retain an integral role in the process of cell differentiation in the chorionic girdle.

Evidence for trophoblast movement was supported by the identification of multiple signalling pathways including focal adhesion, Rho signalling and integrin signalling. Canonical pathway analysis indicated an increase in activity at days 30–31 of pregnancy providing evidence of temporally and spatially regulated trophoblast movement, distinct from invasion of the endometrium which is observed beyond day 34 of development. Signalling by Rho Family of GTPases was the most predominant movement related pathway identified in bioinformatics analysis and functional assays further supported a role for Rho A signalling in equine trophoblast migration. Very little is known about the pathways that regulate movement of chorionic girdle trophoblast, beyond early work that showed a role for metalloproteinases (Vagnoni *et al.* 1995). In human trophoblast, Rho A mediates EGF-regulated migration (Han *et al*. 2010). Based on the induction of EGF in pregnant endometrium in mare pregnancy (Allen *et al.* 2017), it is plausible that this molecular pathway is also important for regulating trophoblast migration in the mare.

Multiple analyses suggested the integrin signalling pathway to be activated increasingly in the chorionic girdle between days 27 and 34 of development. Integrin α2 (*ITGA2*) expression was a top 20 upregulated gene at day 34 consistent with previous work (Sones *et al.* 2010). Regulation of binding of collagen and laminin is a well-defined role of ITGA2 and as these are also known to be expressed in mare endometrium (Mansour *et al.* 2003). Collectively, this is supportive of a role for integrin signalling in invasion of the maternal endometrium around day 36 of pregnancy. Activation of ERK signalling by integrin pathways is thought to play a major role in cell migration, through phosphorylation of myosin chains (Kumar 1998). It is possible that interactions between integrin and ERK signalling is key in co-ordinating the differentiation and migration processes in the chorionic girdle. Whether the trophoblasts migrate to the apices of the girdle folds to differentiate into binucelate eCG-producing cells or they first differentiate and later gain the ability to move towards chemotactic signals is not clear, but this co-ordinated differentiation and cell movement during the development of the chorionic girdle is key and occurs prior to acquisition of invasive potential by trophoblast between days 34 and 38 of pregnancy.

Migration of cancer cells and progression of tumour were identified as some of the top regulator effector networks in the chorionic girdle at all time points. Additionally, the majority of pathways identified in KEGG pathway analysis of gene clusters 1 and 7 were also cancer signalling pathways, with 13 different cancer signalling pathways emerging in total. Trophoblasts have been directly compared to cancer cells in their ability to proliferate, invade the maternal endometrium, establish a blood supply and moderate the maternal immune response (Reeves & James 2017). However, unlike cancer cells, chorionic girdle cells possess an unknown mechanism by which invasion is halted, upon formation of the endometrial cups. Further, chorionic girdle trophoblast senesce upon terminal differentiation, and primary cell cultures have not been maintained much beyond 180 days of culture (Allen & Moor 1972, Thway *et al.* 2001). Investigating how trophoblast cells regulate their invasion may prove highly informative not only in the field of placental biology, but also for identification of novel therapeutic targets in invasive cancers.

A number of canonical pathways involved in immune signalling were identified in analysis of the microarray data. These included CD28 signalling, cluster of differentiation 40 (CD40) signalling and interleukin 9 (IL-9) signalling, all of which peaked in activation at day 34 of pregnancy. Previous work has shown that equine trophoblast cells are able to resist immune destruction when transplanted across allogenic barriers consistent with an inherent ability to modulate the immune response (de Mestre *et al.* 2011). IL-9 secreted by human syncytiotrophoblast have been found to play a role in regulation of integrins to promote cellular migration (Sharma *et al.* 2016). It is therefore possible that a novel role for IL-9 in regulation of endometrial invasion in the equine conceptus through either immunomodulation and/or upregulation of integrin signalling pathways has been identified here. CD40 and CD28 signalling pathways are previously undocumented in placental trophoblast cells of horse, human or mouse origin with further studies required to confirm their activity here. CD40 is commonly found on antigen presenting cells and CD28 on T-helper cells. Upregulation of these signalling pathways would be expected in the immune cells in the maternal endometrium upon invasion by chorionic girdle trophoblasts, and further investigation of the possible activity and function of these pathways in trophoblast is warranted.

In conclusion, through analysis of microarray data using multiple pathway analysis tools, we have been able to generate an overall impression of the genes and signalling landscape in the chorionic girdle within a 7-day period significantly expanding our understanding of molecular networks in equine trophoblast. Functional assays provided further evidence suggesting roles for ELF5 in biochemical differentiation of trophoblast, Rho A in trophoblast migration and ERK1/2 activity that is likely to mediate FGF7 and EGF signalling during chorionic girdle development. Interrogation of pathway components with *in vitro* experimentation will further uncover which of these pathways are crucial for the processes of cell movement, differentiation and invasion not only allowing us to gain a more comprehensive understanding of trophoblast biology in horses but possibly informing future work in other species.

## Supplementary Material

Supporting Figure 1

Supporting Figure 2

Supporting Figure 3

Supporting Figure 4

Supporting Table 1

Supporting Table 2

Supporting Table 3

Supporting Table 4

## Declaration of interest

The authors declare that there is no conflict of interest that could be perceived as prejudicing the impartiality of the research reported.

## Funding

This work was supported by the Wellcome Trust (WT98059 to A M d M and Wbib93257MA to R C F) and Paul Mellon PhD Studentship (to A M d M and R C F).

## References

[bib3] Allen WR Moor RM 1972 The origin of the equine endometrial cups. I. Production of PMSG by fetal trophoblast cells. Journal of Reproduction and Infertility 29 313–316. (10.1530/jrf.0.0290313) 5023705

[bib4] Allen WR Wilsher S 2009 A review of implantation and early placentation in the mare. Placenta 30 1005–1015. (10.1016/j.placenta.2009.09.007) 19850339

[bib1] Allen WR Gower S Wilsher S 2007 Immunohistochemical localization of vascular endothelial growth factor (VEGF) and its two receptors (Flt-I and KDR) in the endometrium and placenta of the mare during the oestrous cycle and pregnancy. Reproduction in Domestic Animals 42 516–526. (10.1111/j.1439-0531.2006.00815.x) 17845608

[bib2] Allen WR Gower S Wilsher S 2017 Localisation of epidermal growth factor (EGF), its specific receptor (EGF-R) and aromatase at the materno-fetal interface during placentation in the pregnant mare. Placenta 50 53–59. (10.1016/j.placenta.2016.12.024) 28161062

[bib5] Antczak DF de Mestre AM Wilsher S Allen WR 2013 The equine endometrial cup reaction: a fetomaternal signal of significance. Annual Review of Animal Biosciences 1 419–442. (10.1146/annurev-animal-031412-103703) 25387026 PMC4641323

[bib6] Bacon SJ Ellis SA Antczak DF 2002 Control of expression of major histocompatibility complex genes in horse trophoblast. Biology of Reproduction 66 1612–1620. (10.1095/biolreprod66.6.1612) 12021038

[bib7] Basak S Sarkar A Mathapati S Duttaroy AK 2018 Cellular growth and tube formation of HTR8/SVneo trophoblast: effects of exogenously added fatty acid-binding protein-4 and its inhibitor Molecular and Cellular Biochemistry 437 55–64 (10.1007/s11010-017-3095-9) 28620819

[bib8] Brosnahan MM Miller DC Adams M Antczak DF 2012 IL-22 is expressed by the invasive trophoblast of the equine (Equus caballus) chorionic girdle. Journal of Immunology 188 4181–4187. (10.4049/jimmunol.1103509) PMC3746837 22490443

[bib9] Brunet A Roux D Lenormand P Dowd S Keyse S Pouyssegur J 1999 Nuclear translocation of p42/p44 mitogen-activated protein kinase is required for growth factor-induced gene expression and cell cycle entry. EMBO Journal 18 664–674. (10.1093/emboj/18.3.664) 9927426 PMC1171159

[bib10] Cabrera-Sharp V Read JE Richardson S Kowalski AA Antczak DF Cartwright JE Mukherjee A de Mestre AM 2014 SMAD1/5 signaling in the early equine placenta regulates trophoblast differentiation and chorionic gonadotropin secretion. Endocrinology 155 3054–3064. (10.1210/en.2013-2116) 24848867 PMC4183921

[bib11] Chen HZ Ouseph MM Li J Pecot T Chokshi V Kent L Bae S Byrne M Duran C Comstock G 2012 Canonical and atypical E2Fs regulate the mammalian endocycle. Nature Cell Biology 14 1192–1202. (10.1038/ncb2595) 23064266 PMC3616487

[bib12] Conesa A Nueda MJ 2017 maSigPro: significant gene expression profile differences in time course gene expression data. R package version 1.52.0. (available at: http://bioinfo.cipf.es/)

[bib13] Costa AF Gomes SZ Lorenzon-Ojea AR Martucci M Faria MR Pinto Ddos S Jr Oliveira SF Letta F Paulesu L Bevilacqua E 2016 Macrophage migration inhibitory factor induces phosphorylation of Mdm2 mediated by phosphatidylinositol 3-kinase/Akt kinase: role of this pathway in decidual cell survival. Placenta 41 27–38. (10.1016/j.placenta.2016.03.001) 27208405

[bib68] de Mestre AM Bacon SJ Costa CC Leadbeater JC Noronha LE Stewart F Antczak DF 2008 Modeling Trophoblast Differentiation using Equine Chorionic Girdle Vesicles. Placenta 29 158–169. (10.1016/j.placenta.2007.10.005) 18054076

[bib15] de Mestre AM Miller D Roberson MS Liford J Chizmar LC McLaughlin KE Antczak DF 2009 Glial cells missing homologue 1 is induced in differentiating equine chorionic girdle trophoblast cells. Biology of Reproduction 80 227–234. (10.1095/biolreprod.108.070920) 18971425 PMC2804814

[bib16] de Mestre AM Noronha LE Wagner B Antczak DF 2010 Split immunological tolerance to trophoblast. International Journal of Developmental Biology 54 445–455. (10.1387/ijdb.082795ad) 19876828 PMC2879498

[bib14] de Mestre AM Hanlon D Adams AP Runcan E Leadbeater JC Erb H Costa C Miller D Allen WR Antczak DF 2011 Functions of ectopically transplanted invasive horse trophoblast. Reproduction 141 849–856. (10.1530/REP-10-0462) 21389079 PMC5181105

[bib17] de Ruijter-Villani M van Boxtel PR Stout TA 2013 Fibroblast growth factor-2 expression in the preimplantation equine conceptus and endometrium of pregnant and cyclic mares. Theriogenology 80 979–989. (10.1016/j.theriogenology.2013.07.024) 24035195

[bib18] Dumaz N Marais R 2005 Integrating signals between cAMP and the RAS/RAF/MEK/ERK signalling pathways. Based on the anniversary prize of the Gesellschaft fur Biochemie und Molekularbiologie Lecture delivered on 5 July 2003 at the special FEBS Meeting in Brussels. FEBS Journal 272 3491–3504. (10.1111/j.1742-4658.2005.04763.x) 16008550

[bib19] Fock V Plessl K Fuchs R Dekan S Milla SK Haider S Fiala C Knofler M Pollheimer J 2015 Trophoblast subtype-specific EGFR/ERBB4 expression correlates with cell cycle progression and hyperplasia in complete hydatidiform moles. Human Reproduction 30 789–799. (10.1093/humrep/dev027) 25740878

[bib20] Gupta SK Malhotra SS Malik A Verma S Chaudhary P 2016 Cell signaling pathways involved during invasion and syncytialization of trophoblast cells. American Journal of Reproductive Immunology 75 361–371. (10.1111/aji.12436) 26490782

[bib21] Haeger JD Hambruch N Pfarrer C 2016 The bovine placenta in vivo and in vitro. Theriogenology 86 306–312. (10.1016/j.theriogenology.2016.04.043) 27155733

[bib22] Han J Li L Hu J Yu J Zheng Y Guo J Zheng X Yi P Zhou Y 2010 Epidermal growth factor stimulates human trophoblast cell migration through Rho A and Rho C activation. Endocrinology 154 1732–1742. (10.1210/en.2009-0845) 20150581

[bib23] Hemberger M Udayashankar R Tesar P Moore H Burton GJ 2010 ELF5-enforced transcriptional networks define an epigenetically regulated trophoblast stem cell compartment in the human placenta. Human Molecular Genetics 19 2456–2467. (10.1093/hmg/ddq128) 20354077

[bib24] Hu X Zhang Y Zhou X Xu B Yang M Wang M Zhang C Li J Bai R Xu W Ma Y 2012 Simultaneously typing nine serotypes of enteroviruses associated with hand, foot, and mouth disease by a GeXP analyzer-based multiplex reverse transcription-PCR assay. Journal of Clinical Microbiology 50 288–293. (10.1128/JCM.05828-11) 22116146 PMC3264198

[bib25] Huang W Sherman BT Lempicki RA 2009 Bioinformatics enrichment tools: paths toward the comprehensive functional analysis of large gene lists. Nucleic Acids Research 37 1–13. (10.1093/nar/gkn923) 19033363 PMC2615629

[bib27] Imakawa K Bai R Fujiwara H Ideta A Aoyagi Y Kusama K 2017 Continuous model of conceptus implantation to the maternal endometrium. Journal of Endocrinology 233 R53–R65. (10.1530/JOE-16-0490) 28213399

[bib28] Iqbal K Chitwood JL Meyers-Brown GA Roser JF Ross PJ 2014 RNA-seq transcriptome profiling of equine inner cell mass and trophectoderm. Biology of Reproduction 90 61. (10.1095/biolreprod.113.113928) 24478389 PMC4435230

[bib29] Jeong W Bazer FW Song G Kim J 2016 Expression of hypoxia-inducible factor-1 by trophectoderm cells in response to hypoxia and epidermal growth factor. Biochemical and Biophysical Research Communications 469 176–182. (10.1016/j.bbrc.2015.11.091) 26620226

[bib30] Klein C Troedsson MH 2011 Transcriptional profiling of equine conceptuses reveals new aspects of embryo-maternal communication in the horse. Biology of Reproduction 84 872–885. (10.1095/biolreprod.110.088732) 21209420

[bib31] Kuang H Chen Q Zhang Y Zhang L Peng H Ning L Cao Y Duan E 2009 The cytokine gene CXCL14 restricts human trophoblast cell invasion by suppressing gelatinase activity. Endocrinology 150 5596–5605. (10.1210/en.2009-0570) 19833716

[bib32] Kumar CC 1998 Signaling by integrin receptors. Oncogene 17 1365–1373. (10.1038/sj.onc.1202172) 9779984

[bib33] Kunath T Yamanaka Y Detmar J MacPhee D Caniggia I Rossant J Jurisicova A 2014 Developmental differences in the expression of FGF receptors between human and mouse embryos. Placenta 35 1079–1088. (10.1016/j.placenta.2014.09.008) 25443433

[bib34] Latos PA Sienerth AR Murray A Senner CE Muto M Ikawa M Oxley D Burge S Cox BJ Hemberger M 2015 Elf5-centered transcription factor hub controls trophoblast stem cell self-renewal and differentiation through stoichiometry-sensitive shifts in target gene networks. Genes and Development 29 2435–2448. (10.1101/gad.268821.115) 26584622 PMC4691948

[bib35] Liang C Park AY Guan J. 2007 *In vitro* scratch assay: a convenient and inexpensive method for analysis of cell migration *in vitro*. Nature Protocols 2 329–333. (10.1038/nprot.2007.30) 17406593

[bib36] MacAuley A Cross JC Werb Z 1998 Reprogramming the cell cycle for endoreduplication in rodent trophoblast cells. Molecular Biology of the Cell 9 795–807. (10.1091/mbc.9.4.795) 9529378 PMC25306

[bib37] Mansour GD Henry M Ferreira AM 2003 Immunohistochemical study of equine endometrial extracellular matrix during the oestrous cycle. Journal of Comparative Pathology 129 316–319. (10.1016/S0021-9975(03)00048-3) 14554131

[bib38] Mattera L Escaffit F Pillaire MJ Selves J Tyteca S Hoffmann JS Gourraud PA Chevillard-Briet M Cazaux C Trouche D 2009 The p400/Tip60 ratio is critical for colorectal cancer cell proliferation through DNA damage response pathways. Oncogene 28 1506–1517. (10.1038/onc.2008.499) 19169279

[bib39] Moffett A Chazara O Colucci F 2017 Maternal allo-recognition of the fetus. Fertility and Sterility 107 1269–1272. (10.1016/j.fertnstert.2017.05.001) 28577615

[bib40] Noronha LE Antczak DF 2010 Maternal immune responses to trophoblast: the contribution of the horse to pregnancy immunology. American Journal of Reproductive Immunology 64 231–244. (10.1111/j.1600-0897.2010.00895.x) 20618178

[bib41] Novoyatleva T Diehl F van Amerongen MJ Patra C Ferrazzi F Bellazzi R Engel FB 2010 TWEAK is a positive regulator of cardiomyocyte proliferation. Cardiovascular Research 85 681–690. (10.1093/cvr/cvp360) 19887380

[bib42] Ohtani K DeGregori J Nevins JR 1995 Regulation of the cyclin E gene by transcription factor E2F1. PNAS 92 12146–12150. (10.1073/pnas.92.26.12146) 8618861 PMC40313

[bib43] Ouseph MM Li J Chen HZ Pecot T Wenzel P Thompson JC Comstock G Chokshi V Byrne M Forde B 2012 Atypical E2F repressors and activators coordinate placental development. Developmental Cell 22 849–862. (10.1016/j.devcel.2012.01.013) 22516201 PMC3483796

[bib44] Pearton DJ Broadhurst R Donnison M Pfeffer PL 2011 Elf5 regulation in the trophectoderm. Developmental Biology 360 343–350. (10.1016/j.ydbio.2011.10.007) 22020251

[bib45] Pearton DJ Smith CS Redgate E van Leeuwen J Donnison M Pfeffer PL 2014 Elf5 counteracts precocious trophoblast differentiation by maintaining Sox2 and 3 and inhibiting Hand1 expression. Developmental Biology 392 344–357. (10.1016/j.ydbio.2014.05.012) 24859262

[bib46] Pfarrer C Weise S Berisha B Schams D Leiser R Hoffmann B Schuler G 2006 Fibroblast growth factor (FGF)-1, FGF2, FGF7 and FGF receptors are uniformly expressed in trophoblast giant cells during restricted trophoblast invasion in cows. Placenta 27 758–770. (10.1016/j.placenta.2005.06.007) 16129484

[bib47] Read JE Cabrera-Sharp V Kitscha P Cartwright JE King PJ Fowkes RC de Mestre AM 2018 Glial cells missing 1 regulates equine chorionic gonadotrophin beta subunit via binding to the proximal promoter. Frontiers in Endocrinology 9 195 (10.3389/fendo.2018.00195) 29755409 PMC5932191

[bib48] Reeves E James E 2017 Tumour and placenta establishment: the importance of antigen processing and presentation. Placenta 56 34–39. (10.1016/j.placenta.2017.02.025) 28274545

[bib49] Ritchie ME Phipson B Wu D Hu Y Law CW Shi W Smyth GK 2015 limma powers differential expression analyses for RNA-sequencing and microarray studies. Nucleic Acids Research 43 e47. (10.1093/nar/gkv007) 25605792 PMC4402510

[bib50] Rosano L Spinella F Bagnato A 2013 Endothelin 1 in cancer: biological implications and therapeutic opportunities. Nature Reviews Cancer 13 637–651. (10.1038/nrc3546) 23884378

[bib51] Shang X Marchioni F Evelyn CR Sipes N Zhou X Seibel W Wortman M Zheng Y 2013 Small-molecule inhibitors targeting G-protein-coupled Rho guanine nucleotide exchange factors. PNAS 110 3155–3160. (10.1073/pnas.1212324110) 23382194 PMC3581902

[bib52] Sharma S Godbole G Modi D 2016 Decidual control of trophoblast invasion. American Journal of Reproductive Immunology 75 341–350. (10.1111/aji.12466) 26755153

[bib53] Sibley CP 2017 Treating the dysfunctional placenta. Journal of Endocrinology 234 R81–R97. (10.1530/JOE-17-0185) 28483805 PMC5516438

[bib54] Soncin F Natale D Parast MM 2015 Signaling pathways in mouse and human trophoblast differentiation: a comparative review. Cellular and Molecular Life Sciences 72 1291–1302. (10.1007/s00018-014-1794-x) 25430479 PMC4366325

[bib55] Sones JL Miller D de Mestre AM Decarlo C Harman R Antczak DF 2010 Investigation of the role of integrin alpha2 subunit in equine trophoblast attachment and invasion. Biology of Reproduction 83 455. (10.1093/biolreprod/83.s1.455)

[bib56] Staines KA Madi K Mirczuk SM Parker S Burleigh A Poulet B Hopkinson M Bodey AJ Fowkes RC Farquharson C 2016 Endochondral growth defect and deployment of transient chondrocyte behaviors underlie osteoarthritis onset in a natural murine model. Arthritis and Rheumatology 68 880–891. (10.1002/art.39508) 26605758 PMC4832379

[bib58] Stewart F Power CA Lennard SN Allen WR Amet L Edwards RM 1994 Identification of the horse epidermal growth factor (EGF) coding sequence and its use in monitoring EGF gene expression in the endometrium of the pregnant mare. Journal of Molecular Endocrinology 12 341–350. (10.1677/jme.0.0120341) 7916972

[bib57] Stewart F Lennard SN Allen WR 1995 Mechanisms controlling formation of the equine chorionic girdle. Biology of Reproduction Monograph 1 151–159.

[bib59] Taniguchi F Harada T Ito M Yoshida S Iwabe T Tanikawa M Terakawa N 2000 Keratinocyte growth factor in the promotion of human chorionic gonadotropin production in human choriocarcinoma cells. American Journal of Obstetrics and Gynecology 182 692–698. (10.1067/mob.2000.104225) 10739532

[bib60] Thway TM Clay CM Maher JK Reed DK McDowell KJ Antczak DF Eckert RL Nilson JH Wolfe MW 2001 Immortalization of equine trophoblast cell lines of chorionic girdle cell lineage by simian virus-40 large T antigen. Journal of Endocrinology 171 45–55. (10.1677/joe.0.1710045) 11572789

[bib61] Underhill LA Robins JC 2016 Trophoblast development in the murine preimplantation embryo. Seminars in Reproductive Medicine 34 57–62. (10.1055/s-0035-1570025) 26757060

[bib62] Vagnoni KE Ginther OJ Lunn DP 1995 Metalloproteinase activity has a role in equine chorionic girdle cell invasion. Biology of Reproduction 53 800–805 (10.1095/biolreprod53.4.800) 8547473

[bib63] Vandesompele J De Preter K Pattyn F Poppe B Van Roy N De Paepe A Speleman F 2002 Accurate normalization of real-time quantitative RT-PCR data by geometric averaging of multiple internal control genes. Genome Biology 3 RESEARCH0034. 12184808 10.1186/gb-2002-3-7-research0034 PMC126239

[bib64] Wang X Miller DC Harman R Antczak DF Clark AG 2013 Paternally expressed genes predominate in the placenta. PNAS 110 10705–10710. (10.1073/pnas.1308998110) 23754418 PMC3696791

[bib65] Wooding FB Morgan G Fowden AL Allen WR 2001 A structural and immunological study of chorionic gonadotrophin production by equine trophoblast girdle and cup cells. Placenta 22 749–767. (10.1053/plac.2001.0707) 11597196

[bib66] Xie L Ichimaru N Morita M Chen J Zhu P Wang J Urbanellis P Shalev I Nagao S Sugioka A 2012 Identification of a novel biomarker gene set with sensitivity and specificity for distinguishing between allograft rejection and tolerance. Liver Transplantation 18 444–454. (10.1002/lt.22480) 22162188

[bib67] Yang QE Giassetti MI Ealy AD 2011 Fibroblast growth factors activate mitogen-activated protein kinase pathways to promote migration in ovine trophoblast cells. Reproduction 141 707–714. (10.1530/REP-10-0541) 21310815

