# Supplementary material for: Dynamic changes in gene expression and signalling during trophoblast development in the horse
Source: Reproduction. 2018 Jul 10;156(4):313–30. doi: 10.1530/REP-18-0270 (PMC6170800; doi:10.1530/REP-18-0270)

Supplementary Figure 1 Representative set of RNA from chorionic girdle and chorion tissues analysed for RNA quality on the Agilent 2100 bioanalyser. (A) A subset of chorionic girdle (ChG) and chorion (CH) RNA samples from all time points (27-34) are shown. Arrows highlight 18S and 28S RNA bands (B) RNA quality was assessed by visualisation of the 18S and 28S bands, measured by area under the peaks.

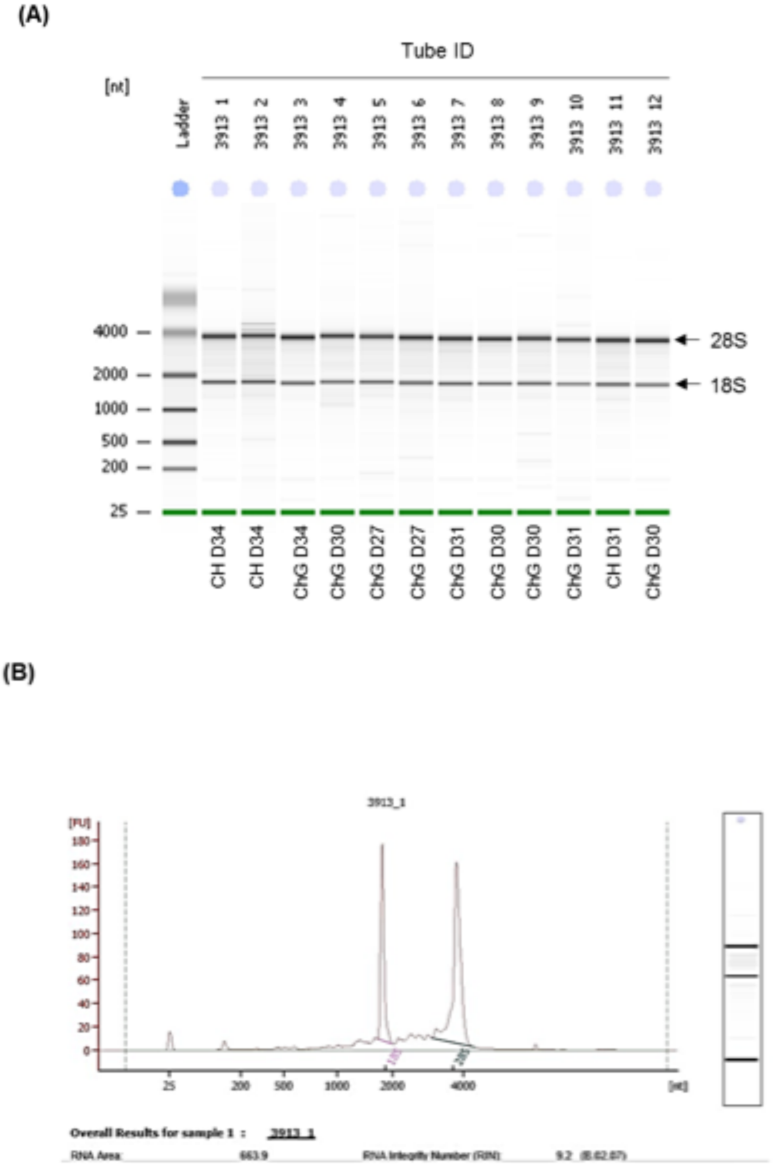

Supplement: Supporting Figure 1 [file rep-156-313-s001.pdf]
