# Supplementary material for: Dynamic changes in gene expression and signalling during trophoblast development in the horse
Source: Reproduction. 2018 Jul 10;156(4):313–30. doi: 10.1530/REP-18-0270 (PMC6170800; doi:10.1530/REP-18-0270)

Supplementary Figure 2: Principle component analysis (PCA) of microarray samples (A) Day 34 chorionic girdle samples clustered clearly away from the other data points with a second cluster containing day 31 and 30 chorionic girdles. Two day 27 samples clustered clearly with those from day 34 conceptuses. (B) Removal of the abnormal mare-stallion pair increased the clustering of the data. (C) Dataset key

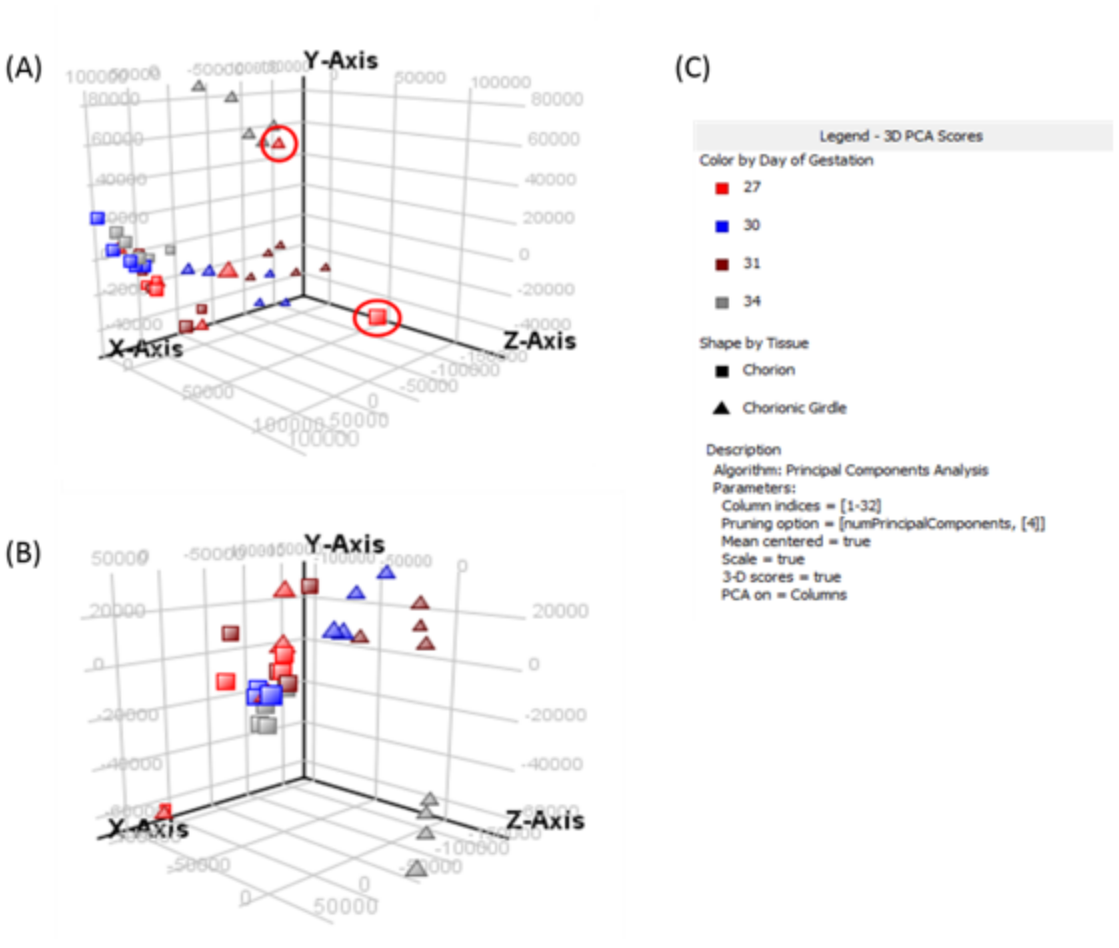

Supplement: Supporting Figure 2 [file rep-156-313-s002.pdf]
