# Supplementary material for: Dynamic changes in gene expression and signalling during trophoblast development in the horse
Source: Reproduction. 2018 Jul 10;156(4):313–30. doi: 10.1530/REP-18-0270 (PMC6170800; doi:10.1530/REP-18-0270)

Supplementary Figure 3 Protein expression of ELF5 in the chorionic girdle. Western blotting of day 34 chorionic girdle (ChG) and Chorion (CH) tissues with an anti-human ELF5 antibody. A faint protein band was seen at approximately 37kDa. (Carried out by Aviva Bioscience, San Diego, US).

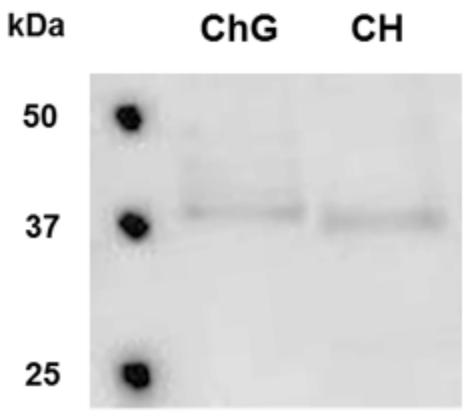

Supplement: Supporting Figure 3 [file rep-156-313-s003.pdf]
