# Supplementary material for: Dynamic changes in gene expression and signalling during trophoblast development in the horse
Source: Reproduction. 2018 Jul 10;156(4):313–30. doi: 10.1530/REP-18-0270 (PMC6170800; doi:10.1530/REP-18-0270)

Supplementary Figure 4 Validation of pCMV-myc-ELF5 expression vector in the COS7 cell line. RNA was extracted from untransfected COS7 cells and COS7 cells transfected with either pCMV-myc- Empty, pCMV-myc-ELF5. RT-PCR was carried out to determine expression of *ELF5* and *CREB3L4*. A H_2O RT-PCR control was run and *B-Actin* PCR carried out as a loading control.

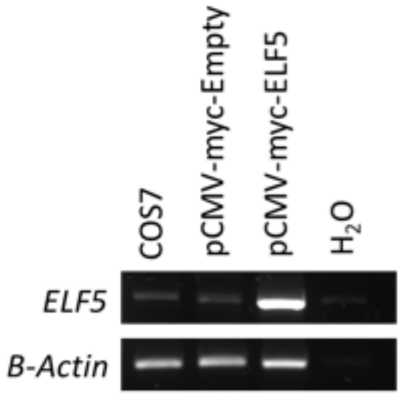

Supplement: Supporting Figure 4 [file rep-156-313-s004.pdf]
