# Supplementary material for: Dynamic changes in gene expression and signalling during trophoblast development in the horse
Source: Reproduction. 2018 Jul 10;156(4):313–30. doi: 10.1530/REP-18-0270 (PMC6170800; doi:10.1530/REP-18-0270)
Supplement: Supporting Table 1 [file rep-156-313-t001.pdf]

Supplementary Table 1 Five mare and stallion pairs were established for breeding purposes. Over a period of two breeding seasons conceptuses were obtained at the four timepoints of interest from each breeding pair. In cases where timepoint samples could not be obtained, tissue sets from other mares, bred with the matched stallion were used to minimise genetic variation (highlighted in yellow).

		Sample set Number			
**Mare**	**Stallion**	**Day 27**	**Day 30**	**Day 31**	**Day 34**
M1	S1	1213	1209	1204	1305
M2	S1	1214	1311	1205	1303
M3	S1	1307	1203	1310	1216
M4	S2	1201	1210	1207	1304
M5	S2	1306	1309	1212	1211
