# Supplementary material for: Dynamic changes in gene expression and signalling during trophoblast development in the horse
Source: Reproduction. 2018 Jul 10;156(4):313–30. doi: 10.1530/REP-18-0270 (PMC6170800; doi:10.1530/REP-18-0270)
Supplement: Supporting Table 2 [file rep-156-313-t002.pdf]

Supplementary Table 2: Primer sequences for multiplex qRTPCR

Gene	Product Size	Forward Primer	Reverse Primer
CREB3L4	137	AGGTGACACTATAGAATACCAGCTGTAGAGCCCATCTC	GTACGACTCACTATAGGGACATGAGCATCAAGGTGCAGT
NKX2-5	144	AGGTGACACTATAGAATAACAACTTCGTGAACTTCGGC	GTACGACTCACTATAGGGACTACCAGGCTCGGATACCGT
GAPDH	151	AGGTGACACTATAGAATATCCTGGGCTACACTGAGGAC	GTACGACTCACTATAGGGATGAGCTTGACAAAGTGGTCG
FOXP1	165	AGGTGACACTATAGAATAGCCTAAAGAGCAACAGCAGG	GTACGACTCACTATAGGGACCGGCTGAATTGTTAGAAGG
CYP7A1	172	AGGTGACACTATAGAATATGACAAAATCTTTCCAGCCC	GTACGACTCACTATAGGGAACCAGTTCCGAGATGTGGTC
SMAD7	179	AGGTGACACTATAGAATACGCTGTTGGTACACAAGGTG	GTACGACTCACTATAGGGATTCACGAAGCTGATCTGCAC
PAX6	186	AGGTGACACTATAGAATACAATCAAAACGTGTCCAACG	GTACGACTCACTATAGGGAACTCCCGCTTATACTGGGCT
S100A12	193	AGGTGACACTATAGAATAAGATGGCCAGGTCAGCTTTA	GTACGACTCACTATAGGGAGTCTGGGCTTTGATGAGGAG
KLF10	200	AGGTGACACTATAGAATAGACCGATTGGAAGGTGAAGA	GTACGACTCACTATAGGGAGCTTGTCATCTAACCCAGGC
SDHA	207	AGGTGACACTATAGAATAGGGAACATGGAAGAGGACAA	GTACGACTCACTATAGGGAATCCTCCCATCTTCGGTTCT
ELF5	214	AGGTGACACTATAGAATAGATGTGGGGACAGAGGAAGA	GTACGACTCACTATAGGGACTTGATGACGGAGCAGATCA
SREBF1	221	AGGTGACACTATAGAATACACTCGTCTTCCTCTGCCTC	GTACGACTCACTATAGGGATTAGTCAGCCAGATCAGGGG
NXF1	229	AGGTGACACTATAGAATAGCGTGGCTCCTAAGTGTGAT	GTACGACTCACTATAGGGAGGAACAGAGGCGTTGATGAT
SRF	236	AGGTGACACTATAGAATAACGACCTTCAGCAAGAGGAA	GTACGACTCACTATAGGGAGAGAGTCTGGCGAGTTGAGG
CREB1	243	AGGTGACACTATAGAATAGAATGTTCCAACACCTGCCT	GTACGACTCACTATAGGGATGTCCCTAAGGCAATCAAGG
ELF4	250	AGGTGACACTATAGAATACCAGTTTAAGGAGATGCCCA	GTACGACTCACTATAGGGATGAAGACCAACGTGCTGAAC
KAN	325	AGGTGACACTATAGAATAATCATCAGCATTGCATTCGATTCCTGTTTG	GTACGACTCACTATAGGGAATTCCGACTCGTCCAACATC
